# Supplementary material for: Distilling noise characteristics and prior expectations in multisensory causal inference
Source: PLoS Comput Biol. 2026 May 8;22(5):e1014251. doi: 10.1371/journal.pcbi.1014251 (PMC13155690; doi:10.1371/journal.pcbi.1014251)
Supplement: S1 Appendix — (PDF) [file pcbi.1014251.s001.pdf]

Distilling noise characteristics and prior expectations in multisensory causal inference

Shuze Liu[1◕], Trevor Holland[2◕], Wei Ji Ma[2,3 &], Luigi Acerbi[4 &*]

1 PhD Program in Neuroscience, Harvard University, Cambridge, Massachusetts, United States of America
2 Previously at Department of Neuroscience, Baylor College of Medicine, Houston, Texas, United States of America, where the experiment and initial analyses were conducted.
3 Center for Neural Science and Department of Psychology, New York University, New York City, New York, United States of America
4 Department of Computer Science, University of Helsinki, Helsinki, Uusimaa, Finland

◕Primary authorship equal contribution
& Senior authorship equal contribution
* luigi.acerbi@helsinki.fi

Supporting information

S1 Appendix

A Lapse distribution selection **2**

B Model comparison result tables **5**
B.1 Unisensory data fits 5
B.1.1 Const-SingleGaussian (vanilla) models 5
B.1.2 All models 7
B.2 All-tasks fits 9

C Model response distributions **13**
C.1 Unisensory parametric model response distributions 13
C.2 Lifted-semiparametric model response distributions 18
C.3 All-tasks parametric model response distributions 22

D Model response distributions, individual participants **24**
D.1 Unisensory data fit 24
D.2 All-tasks fit 27
D.3 Individual fitted parameters 32

Section A Lapse distribution selection

One observation in all our model fit visualizations over unisensory data is a systematic overestimation of the SD in participant responses. This is demonstrated in Subpanel c) of every relevant figure starting from Fig 2 in the main text. This SD overestimation is a consequence of our simplistic lapse distribution assumption, which gives nonzero probabilities of responding uniformly within the response range $[-45, 45]$, yielding possibly unrealistically large responses.

For demonstration purposes, we have refrained from plotting lapses in Fig A. Similar to Fig 8 in the main text, this figure portrays the Exp-GaussianLaplace model fitted to unisensory data, but now without showing lapse responses in the model predictive samples. A comparison between Fig 8C in the main text and Fig A Panel C here demonstrates how the uniform lapse leads to SD overestimations.

In light of this SD overestimation effect, we have also considered a truncated-Gaussian lapse distribution with the response range $[-45, 45]$ to mitigate the problem. In contrast to the uniform lapse distribution $U[-45, 45]$ used in all previous models, the truncated-Gaussian lapse distribution requires the fitting of not only a lapse rate λ, but also the lapse distribution's standard deviation σ_{lapse}.

We have fitted an Exp-GaussianLaplace model assuming Gaussian-lapse to the unisensory data. Unfortunately, its visualization in Fig B reveals that using a truncated-Gaussian lapse distribution does not seem to resolve the SD overestimation problem. Furthermore, BIC model comparison results cannot distinguish the truncated-Gaussian lapse model from its uniform lapse counterpart featuring one less parameter. Specifically, the Sum and $[2.5\%, 97.5\%]$ bootstrapping intervals for the BIC difference are -41.8 and $[-114, 18.6]$ respectively, where the difference is computed with respect to the Uniform lapse model. For this reason, we used the standard uniform lapse model in the main text, leaving further investigations for future work.

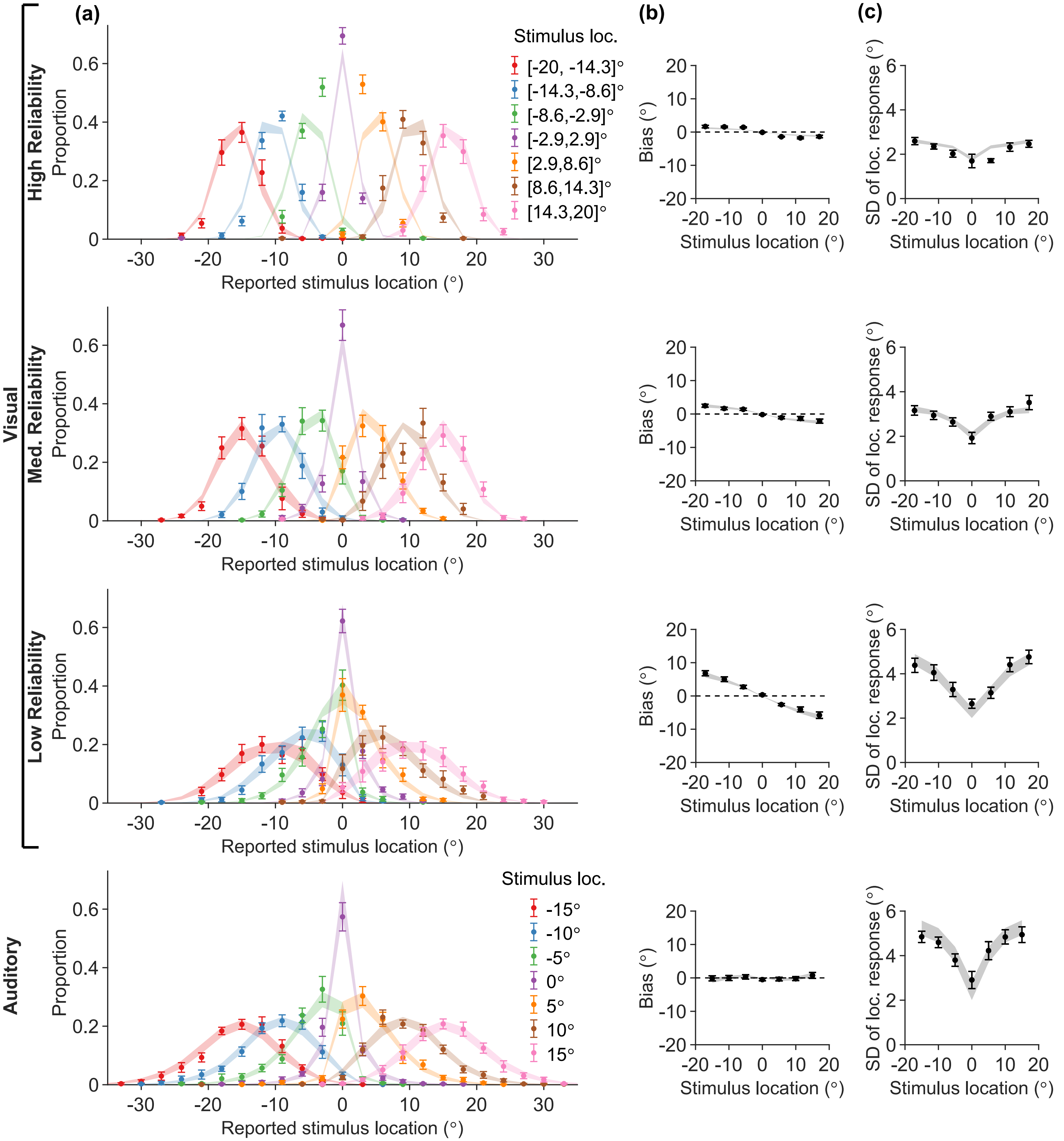

Fig A. The Exp-GaussianLaplace parametric model fitted jointly on UV and UA data for all participants, visualized without lapses. This figure is identical to Fig 8 in the main text, but without lapses in the model predictive samples. The layout and color schemes are identical to Fig 8 in the main text.

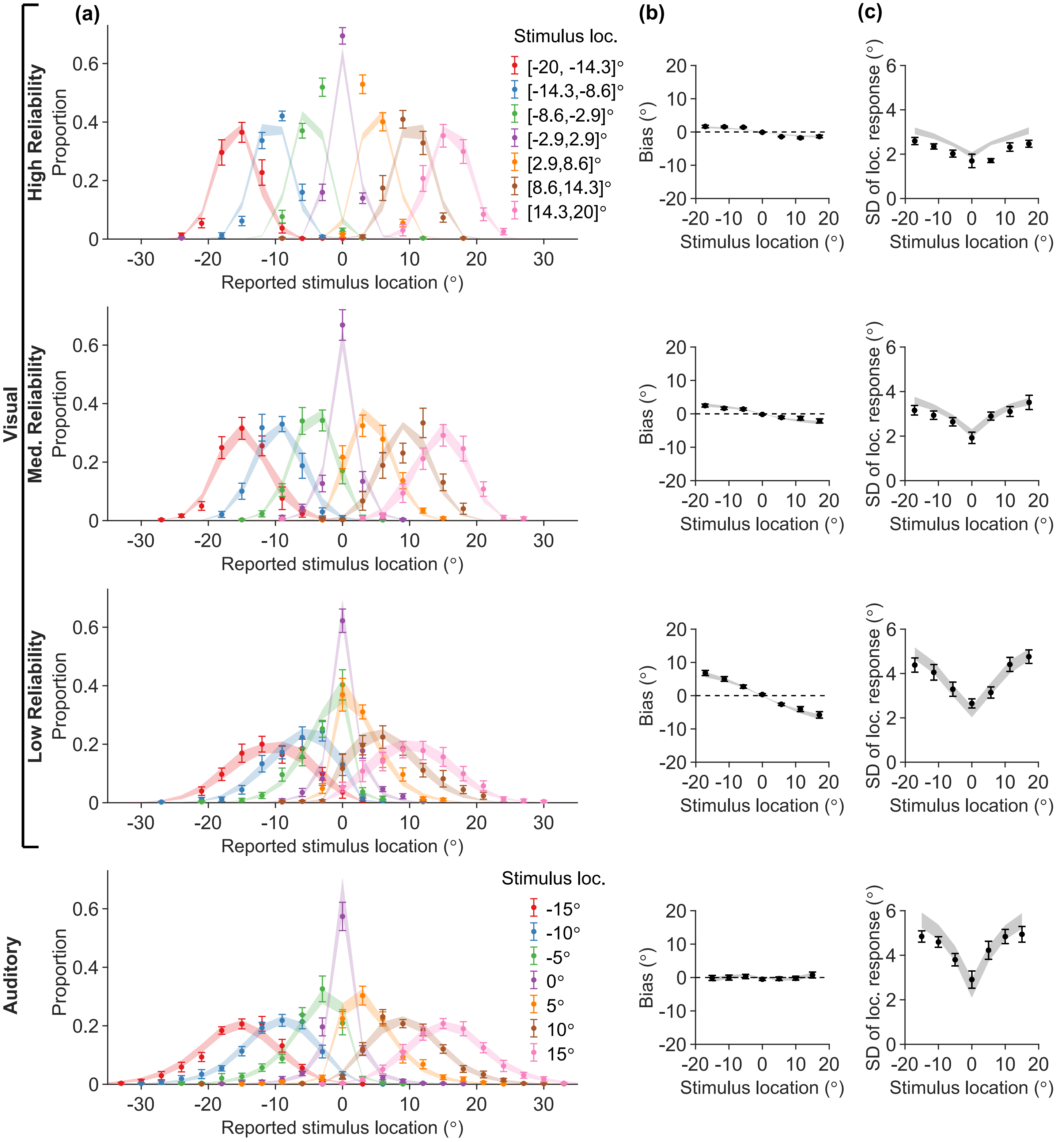

Fig B. The Exp-GaussianLaplace parametric model fitted jointly on UV and UA data for all participants, in which the auditory range recalibration factor is a free parameter, and a Gaussian lapse distribution is used. The layout and color schemes are identical to Fig A.

Section B Model comparison result tables

Section B.1 Unisensory data fits

Section B.1.1 Const-SingleGaussian (vanilla) models

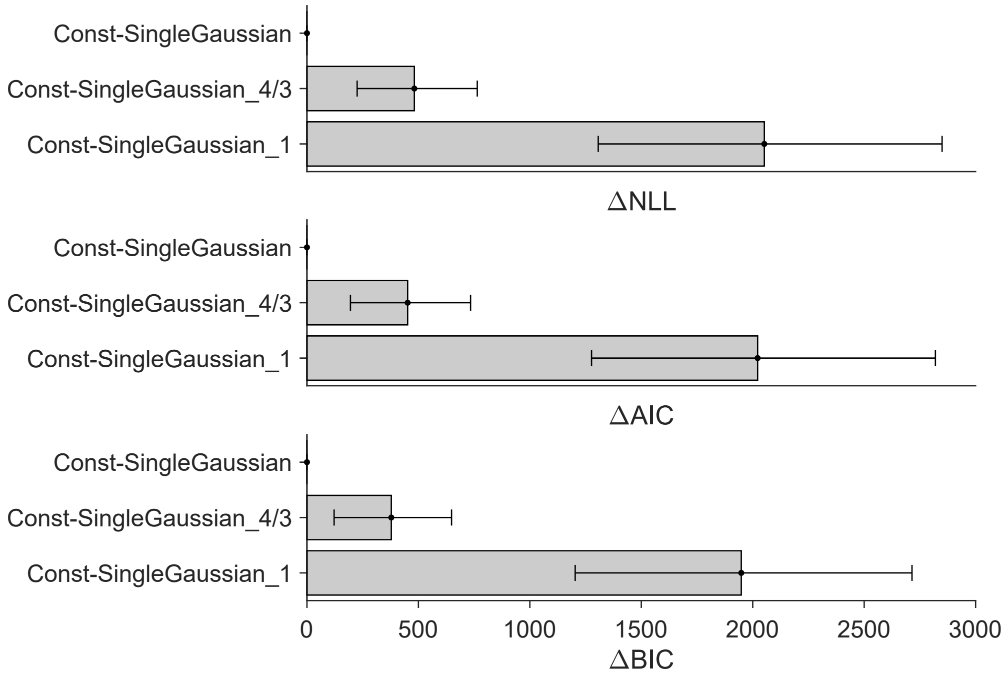

Fig C. NLL, AIC, and BIC model comparison results for Const-SingleGaussian (vanilla) parametric models fitted on UV+UA data. Quantitative values are in the tables below.

Table A. NLL differences of Const-SingleGaussian (vanilla) models fitted on unisensory data, with sum-across-participants values and 95% bootstrapping intervals.

	Sum	CI (2.5%, 97.5%)
Const-SingleGaussian	0	(0, 0)
Const-SingleGaussian_4/3	240.90	(112.56, 382.04)
Const-SingleGaussian_1	1026.00	(653.40, 1424.77)

Table B. AIC differences of Const-SingleGaussian (vanilla) models fitted on unisensory data, with sum-across-participants values and 95% bootstrapping intervals.

	Sum	CI (2.5%, 97.5%)
Const-SingleGaussian	0	(0, 0)
Const-SingleGaussian_4/3	451.80	(195.11, 734.08)
Const-SingleGaussian_1	2022.00	(1276.81, 2819.54)

Table C. BIC differences of Const-SingleGaussian (vanilla) models fitted on unisensory data, with sum-across-participants values and 95% bootstrapping intervals.

	Sum	CI (2.5%, 97.5%)
Const-SingleGaussian	0	(0, 0)
Const-SingleGaussian_4/3	378.42	(121.79, 648.75)
Const-SingleGaussian_1	1948.62	(1203.43, 2714.57)

Section B.1.2 All models

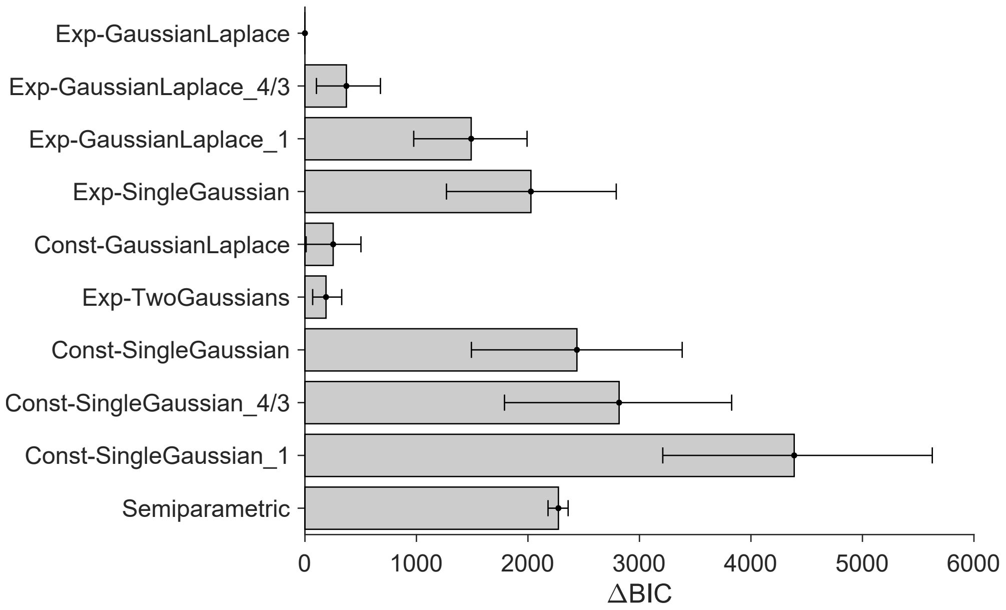

Fig D. BIC model comparison results for models fitted on UV+UA data. Quantitative values are in the tables below.

Table D. NLL differences of all models fitted on unisensory data, with sum-across-participants values and bootstrapping intervals.

	Sum	CI (2.5%, 97.5%)
Semiparametric	0	(0, 0)
Exp-GaussianLaplace	207.35	(163.56, 252.06)
Exp-GaussianLaplace_4/3	444.93	(293.59, 629.90)
Exp-GaussianLaplace_1	1004.52	(751.52, 1264.55)
Exp-SingleGaussian	1324.08	(916.25, 1750.00)
Const-GaussianLaplace	540.74	(383.44, 705.77)
Exp-TwoGaussians	301.86	(213.96, 396.62)
Const-SingleGaussian	1737.16	(1235.21, 2257.05)
Const-SingleGaussian_4/3	1978.06	(1432.95, 2534.14)
Const-SingleGaussian_1	2763.16	(2150.18, 3426.67)

Table E. AIC differences of models fitted on unisensory data, with sum-across-participants values and bootstrapping intervals.

	Sum	CI (2.5%, 97.5%)
Semiparametric	365.31	(275.87, 452.88)
Exp-GaussianLaplace	0	(0, 0)
Exp-GaussianLaplace_4/3	445.18	(176.60, 763.97)
Exp-GaussianLaplace_1	1564.36	(1049.02, 2088.37)
Exp-SingleGaussian	2173.48	(1416.30, 2971.41)
Const-GaussianLaplace	546.79	(304.72, 805.99)
Exp-TwoGaussians	189.03	(69.30, 336.71)
Const-SingleGaussian	2879.62	(1934.28, 3862.44)
Const-SingleGaussian_4/3	3331.42	(2304.28, 4382.25)
Const-SingleGaussian_1	4901.63	(3723.83, 6196.64)

Table F. BIC differences of models fitted on unisensory data, with sum-across-participants values and bootstrapping intervals.

	Sum	CI (2.5%, 97.5%)
Semiparametric	2273.21	(2180.21, 2360.43)
Exp-GaussianLaplace	0	(0, 0)
Exp-GaussianLaplace_4/3	371.80	(103.23, 677.21)
Exp-GaussianLaplace_1	1490.98	(975.64, 1992.09)
Exp-SingleGaussian	2026.72	(1269.31, 2791.96)
Const-GaussianLaplace	253.27	(10.68, 502.29)
Exp-TwoGaussians	189.03	(69.30, 329.82)
Const-SingleGaussian	2439.34	(1492.82, 3383.42)
Const-SingleGaussian_4/3	2817.76	(1789.63, 3827.16)
Const-SingleGaussian_1	4387.96	(3209.29, 5626.36)

Section B.2 All-tasks fits

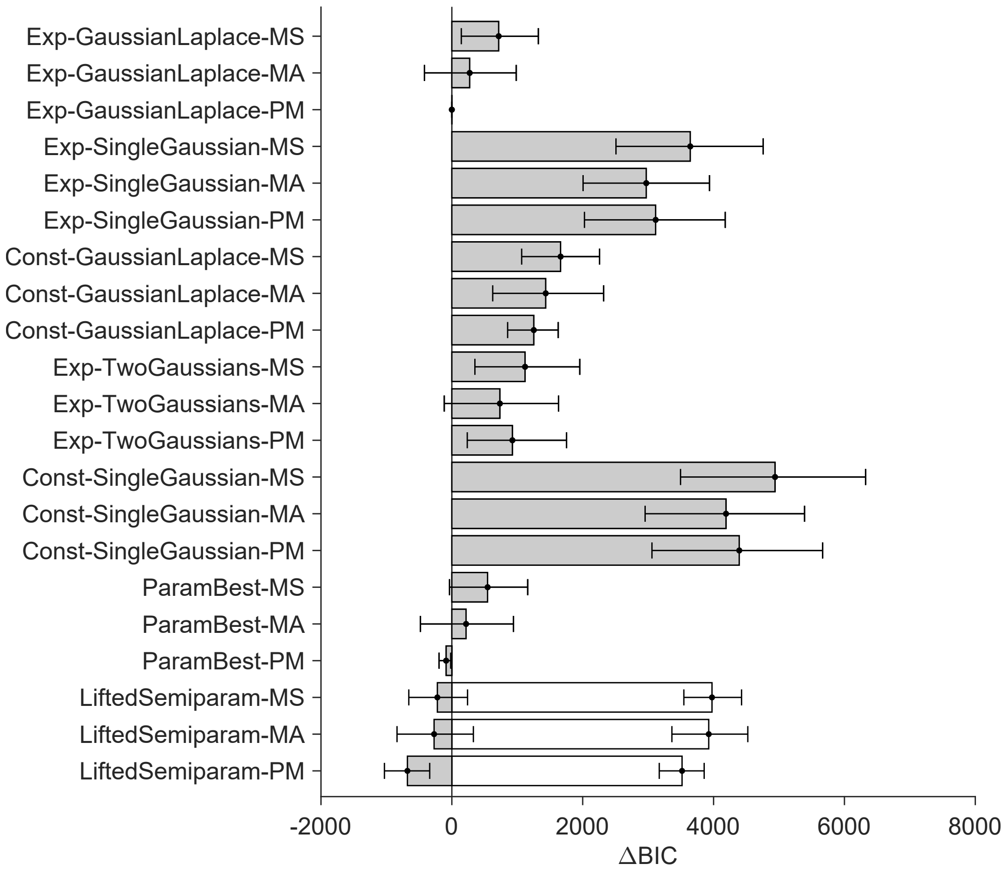

Fig E. BIC model comparison results for parametric models fitted on all tasks. For the LiftedSemiparam models, the white bar denotes BIC scores accounting for parameters fitted in the earlier semiparametric fits that LiftedSemiparam model fits were based on. The gray bar denotes BIC scores that exclude these parameters. Quantitative values are in the tables below.

Table G. NLL differences of models fitted on all tasks, with sum-across-participants values and bootstrapping intervals.

	Sum	CI (2.5%, 97.5%)
Exp-GaussianLaplace-MS	357.80	(72.31, 674.7)
Exp-GaussianLaplace-MA	136.10	(-209.48, 507.23)
Exp-GaussianLaplace-PM	0	(0, 0)
Exp-SingleGaussian-MS	1941.64	(1374.28, 2522.51)
Exp-SingleGaussian-MA	1605.36	(1123.01, 2109.31)
Exp-SingleGaussian-PM	1677.29	(1133.31, 2230.46)
Const-GaussianLaplace-MS	1070.72	(773.87, 1381.42)
Const-GaussianLaplace-MA	956.96	(552.64, 1419.1)
Const-GaussianLaplace-PM	865.79	(666.54, 1059.77)
Exp-TwoGaussians-MS	559.20	(176.21, 996.49)
Exp-TwoGaussians-MA	366.71	(-58.13, 836.76)
Exp-TwoGaussians-PM	462.45	(117.84, 896.98)
Const-SingleGaussian-MS	2829.18	(2107.87, 3548.45)
Const-SingleGaussian-MA	2454.61	(1837.34, 3077.97)
Const-SingleGaussian-PM	2555.92	(1889.36, 3217.92)
ParametricBest-MS	273.13	(-17.98, 593.01)
ParametricBest-MA	116.55	(-233.08, 493.42)
ParametricBest-PM	-43.32	(-97.43, -7.55)
LiftedSemiparametric-MS	368.95	(151.54, 610.39)
LiftedSemiparametric-MA	344.07	(61.09, 657.78)
LiftedSemiparametric-PM	140.17	(-35.01, 317.72)

Table H. AIC differences of models fitted on all tasks, with sum-across-participants values and bootstrapping intervals. * denotes AIC computed for LiftedSemiparam models, including parameters fitted during the earlier Semiparam model fits.

	Sum	CI (2.5%, 97.5%)
Exp-GaussianLaplace-MS	715.59	(144.62, 1349.4)
Exp-GaussianLaplace-MA	272.19	(-418.95, 1014.46)
Exp-GaussianLaplace-PM	0	(0, 0)
Exp-SingleGaussian-MS	3823.28	(2688.56, 4985.02)
Exp-SingleGaussian-MA	3150.72	(2186.02, 4158.63)
Exp-SingleGaussian-PM	3294.57	(2206.63, 4400.92)
Const-GaussianLaplace-MS	2021.44	(1427.74, 2642.83)
Const-GaussianLaplace-MA	1793.93	(985.29, 2718.2)
Const-GaussianLaplace-PM	1611.58	(1213.09, 1999.54)
Exp-TwoGaussians-MS	1118.40	(352.41, 1992.97)
Exp-TwoGaussians-MA	733.41	(-116.26, 1673.52)
Exp-TwoGaussians-PM	924.91	(235.68, 1793.97)
Const-SingleGaussian-MS	5478.35	(4035.73, 6916.9)
Const-SingleGaussian-MA	4729.21	(3494.69, 5975.95)
Const-SingleGaussian-PM	4931.83	(3598.72, 6255.85)
ParametricBest-MS	546.25	(-35.95, 1186.02)
ParametricBest-MA	229.10	(-469.63, 982.62)
ParametricBest-PM	-86.64	(-194.86, -15.1)
LiftedSemiparametric-MS	497.90	(63.08, 980.78)
LiftedSemiparametric-MA	448.14	(-117.81, 1075.57)
LiftedSemiparametric-PM	40.34	(-310.02, 395.43)
LiftedSemiparametric-MS*	1547.90	(1113.08, 2030.78)
LiftedSemiparametric-MA*	1498.14	(932.19, 2125.57)
LiftedSemiparametric-PM*	1090.34	(739.98, 1445.43)

Table I. BIC differences of models fitted on all tasks, with sum-across-participants values and bootstrapping intervals. * denotes BIC computed for LiftedSemiparam models, including parameters fitted during the earlier Semiparam model fits.

	Sum	CI (2.5%, 97.5%)
Exp-GaussianLaplace-MS	715.59	(144.62, 1321.81)
Exp-GaussianLaplace-MA	272.19	(-418.95, 984.14)
Exp-GaussianLaplace-PM	0	(0, 0)
Exp-SingleGaussian-MS	3643.42	(2508.39, 4758.8)
Exp-SingleGaussian-MA	2970.86	(2005.75, 3937.04)
Exp-SingleGaussian-PM	3114.71	(2026.6, 4178.73)
Const-GaussianLaplace-MS	1661.72	(1067.5, 2257.67)
Const-GaussianLaplace-MA	1434.20	(624.75, 2320.52)
Const-GaussianLaplace-PM	1251.86	(852.92, 1625.8)
Exp-TwoGaussians-MS	1118.40	(352.41, 1955.4)
Exp-TwoGaussians-MA	733.41	(-116.26, 1631.71)
Exp-TwoGaussians-PM	924.91	(235.68, 1753.44)
Const-SingleGaussian-MS	4938.77	(3495.05, 6323.04)
Const-SingleGaussian-MA	4189.63	(2953.93, 5389.78)
Const-SingleGaussian-PM	4392.25	(3058.31, 5666.54)
ParametricBest-MS	546.25	(-35.95, 1159.76)
ParametricBest-MA	217.08	(-481.21, 940.81)
ParametricBest-PM	-86.64	(-194.86, -17.57)
LiftedSemiparametric-MS	-221.55	(-657.97, 240.25)
LiftedSemiparametric-MA	-271.30	(-837.84, 328.91)
LiftedSemiparametric-PM	-679.10	(-1030.17, -338.32)
LiftedSemiparametric-MS*	3976.02	(3545.92, 4427.55)
LiftedSemiparametric-MA*	3926.26	(3363.12, 4523.31)
LiftedSemiparametric-PM*	3518.46	(3170.21, 3856.51)

Section C Model response distributions

Section C.1 Unisensory parametric model response distributions

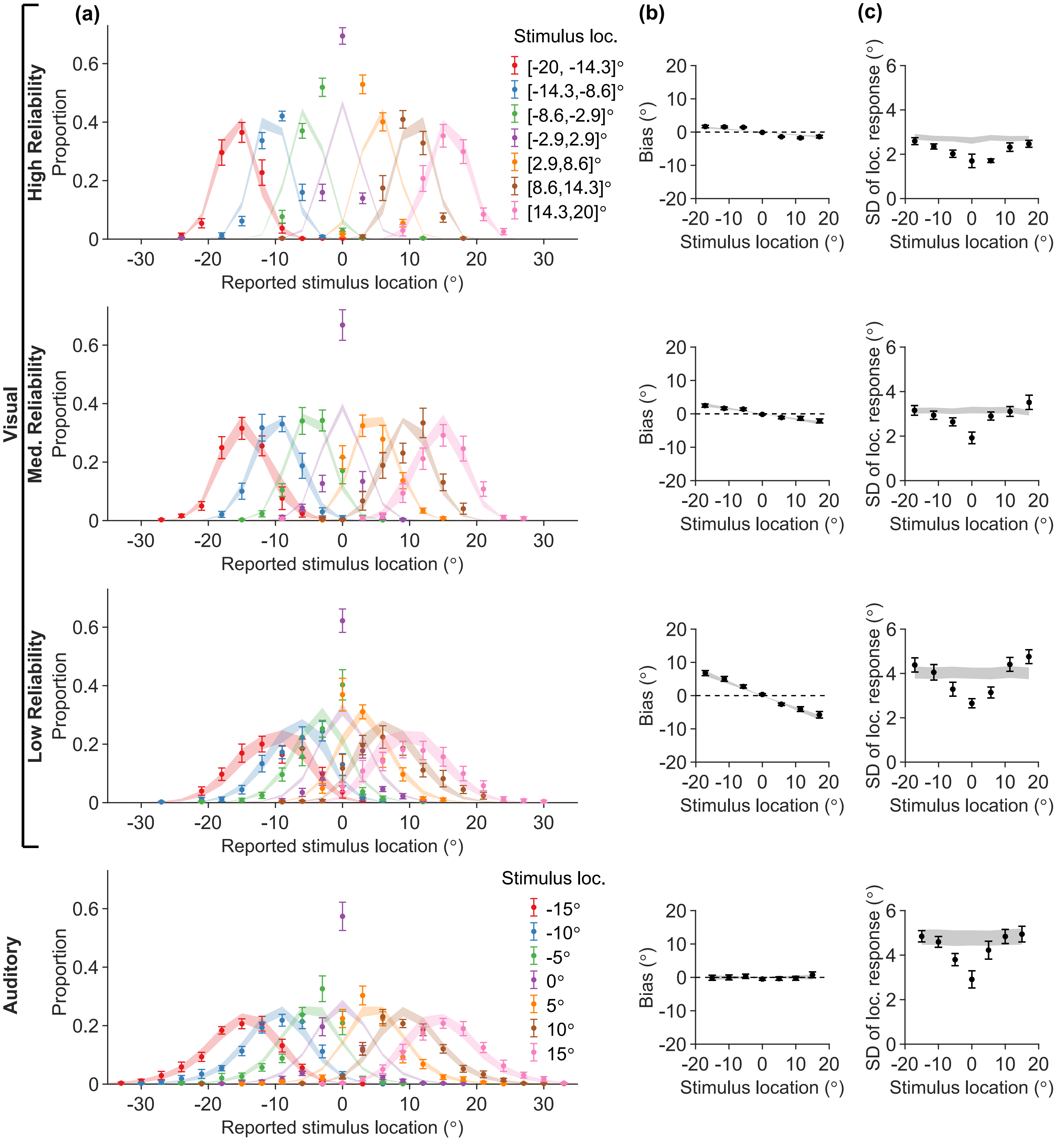

Fig F. The Const-SingleGaussian (vanilla) model fitted jointly on UV and UA data for all participants, with a free auditory range recalibration parameter. Subplot notations are identical to Fig A.

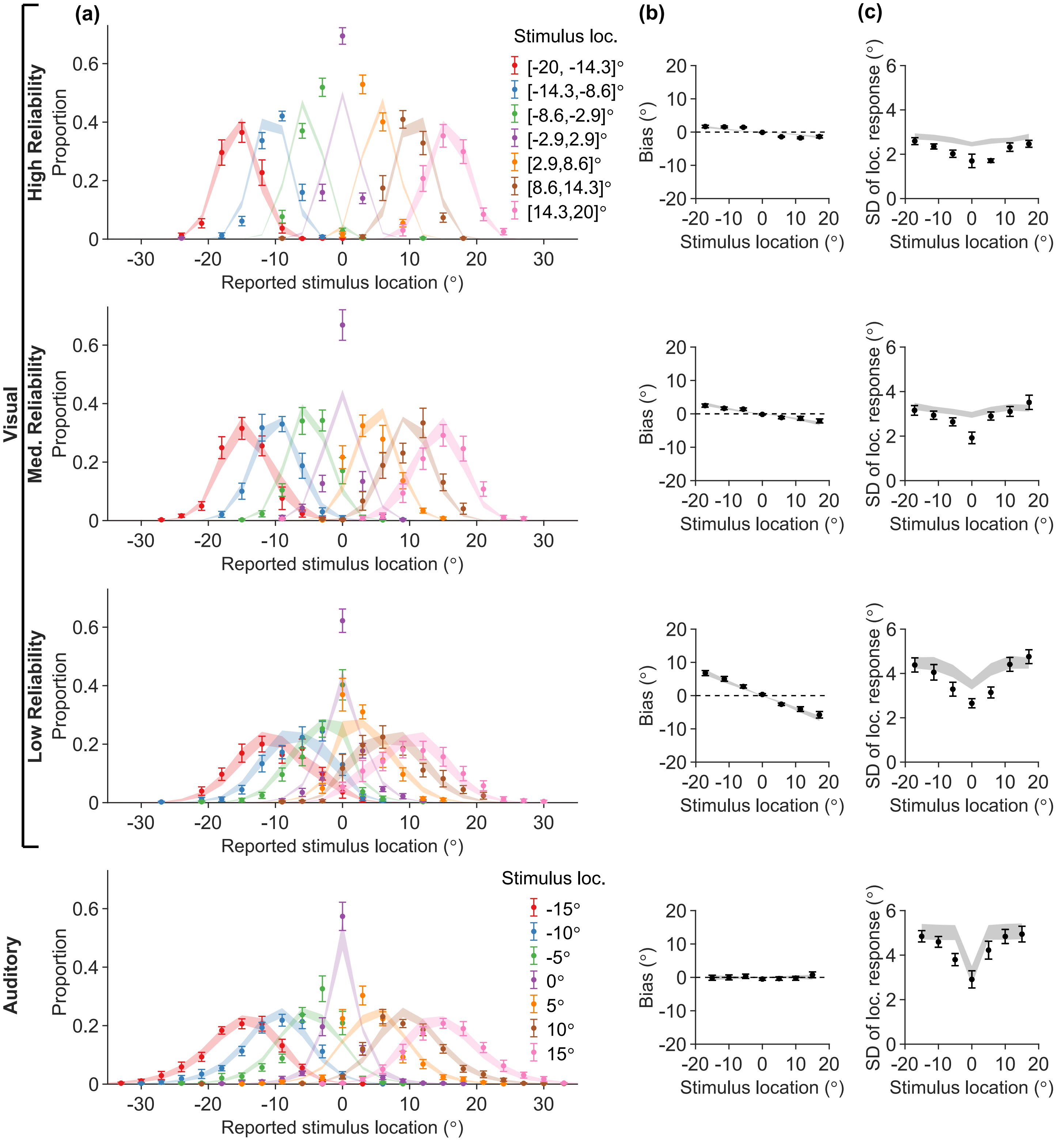

Fig G. The Exp-SingleGaussian parametric model fitted jointly on UV and UA data for all participants, in which the auditory range recalibration factor is a free parameter. The layout and color schemes are identical to Fig A.

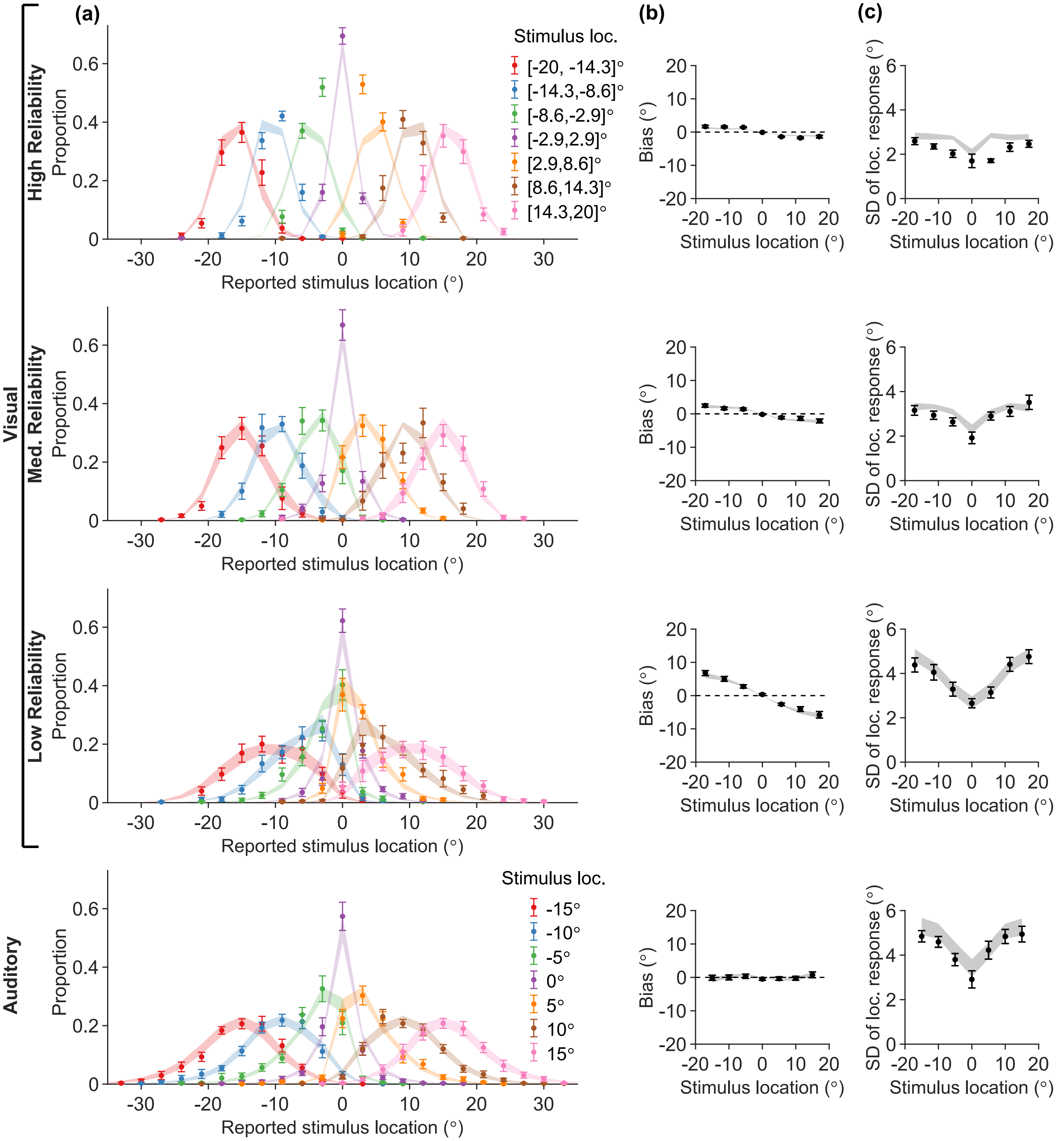

Fig H. The Const-GaussianLaplace parametric model fitted jointly on UV and UA data for all participants, in which the auditory range recalibration factor is a free parameter. The layout and color schemes are identical to Fig A.

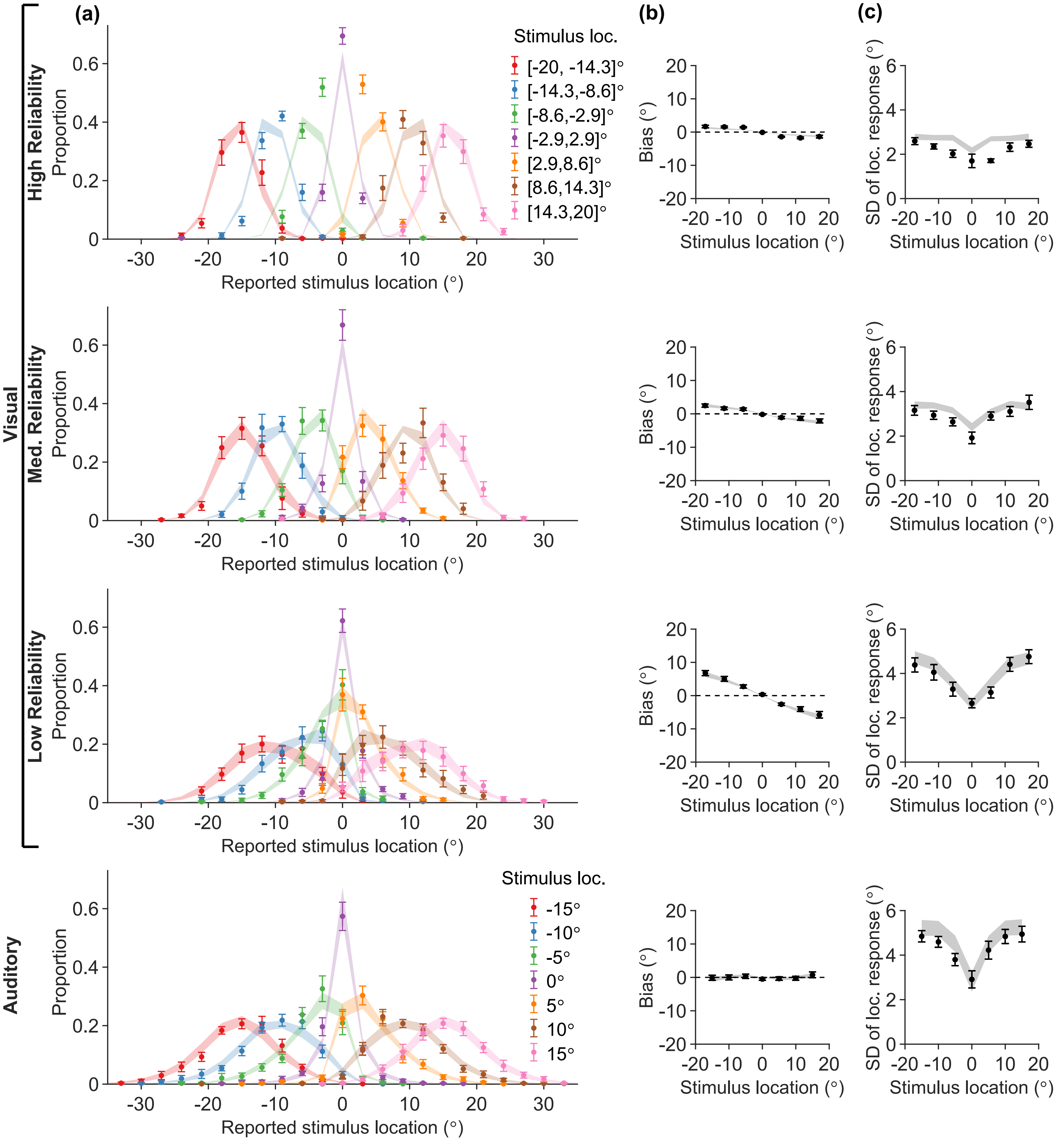

Fig I. The Exp-TwoGaussians parametric model fitted jointly on UV and UA data for all participants, in which the auditory range recalibration factor is a free parameter. The layout and color schemes are identical to Fig A.

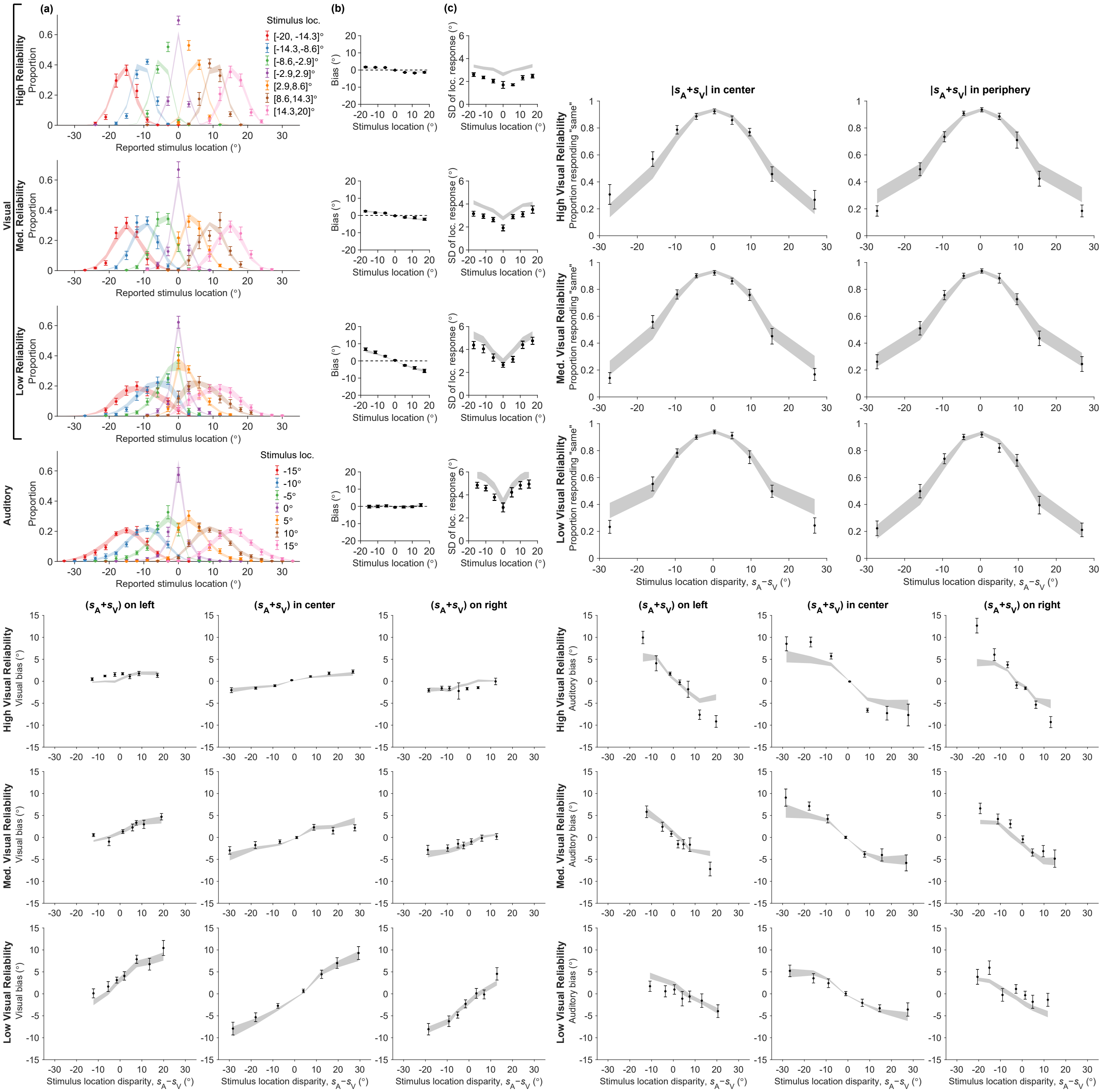

Fig J. The lifted-semiparametric fits on all tasks, assuming the MS causal inference strategy. (A) UV+UA response distributions from data and model predictive samples. (B) BC response distributions from data and model predictive samples. Rows correspond to different visual reliability levels, and Cols are stratified based on whether the sum of the two true stimuli locations is below or above its median value across all BC trials. (C) BV response distributions from data and model predictive samples. Rows correspond to different visual reliability levels, and Cols are stratified based on where the sum of the two true stimuli locations lies with respect to its 100/3th and 200/3 percentiles across all BV trials. (D) BA response distributions from data and model predictive samples. Rows correspond to different visual reliability levels, and Cols are stratified based on where the sum of the two true stimuli locations lies with respect to its 100/3th and 200/3 percentiles across all BA trials.

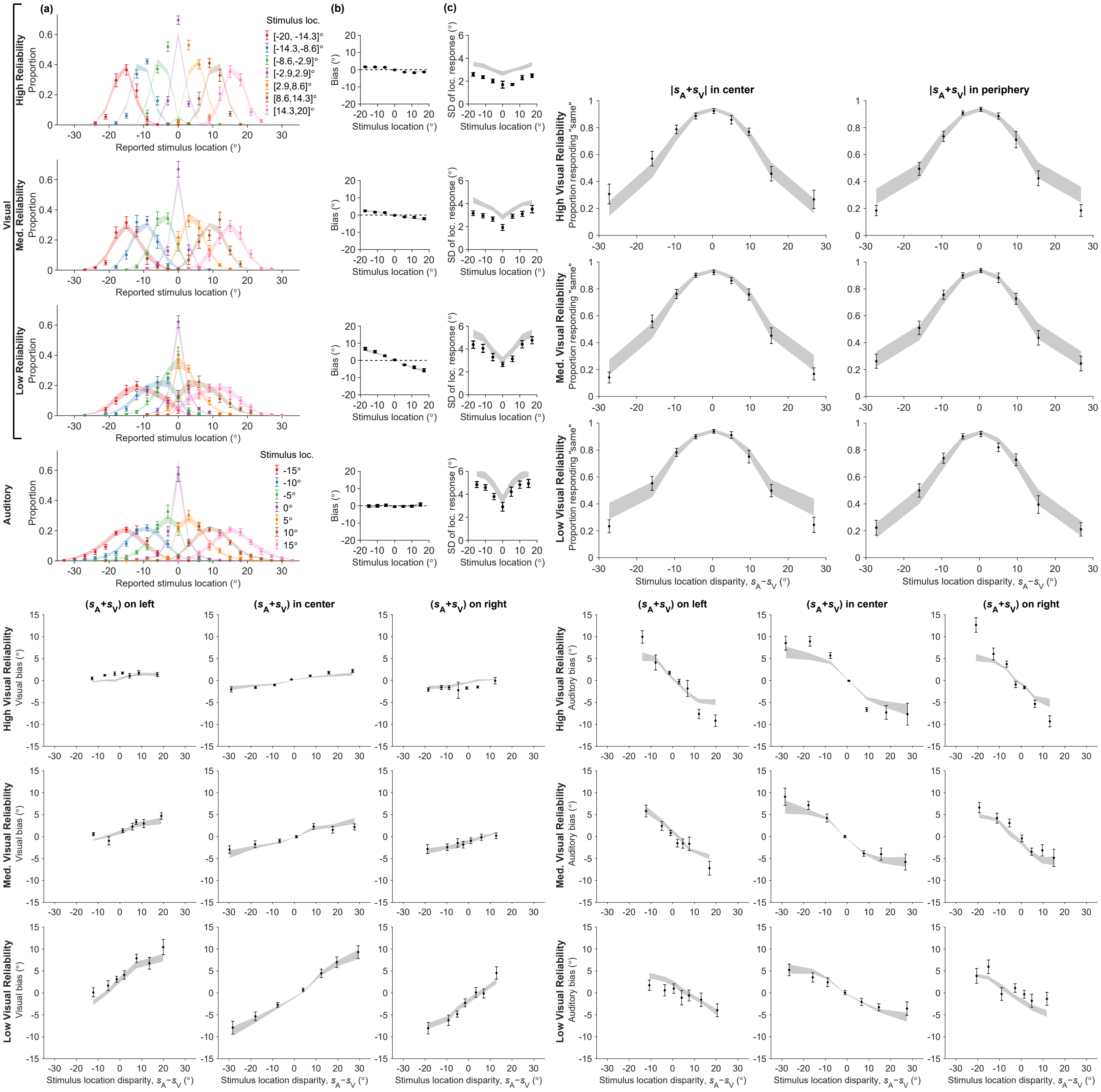

Fig K. The lifted-semiparametric fits on all tasks, assuming the MA causal inference strategy. Subplot legends are identical to Fig J.

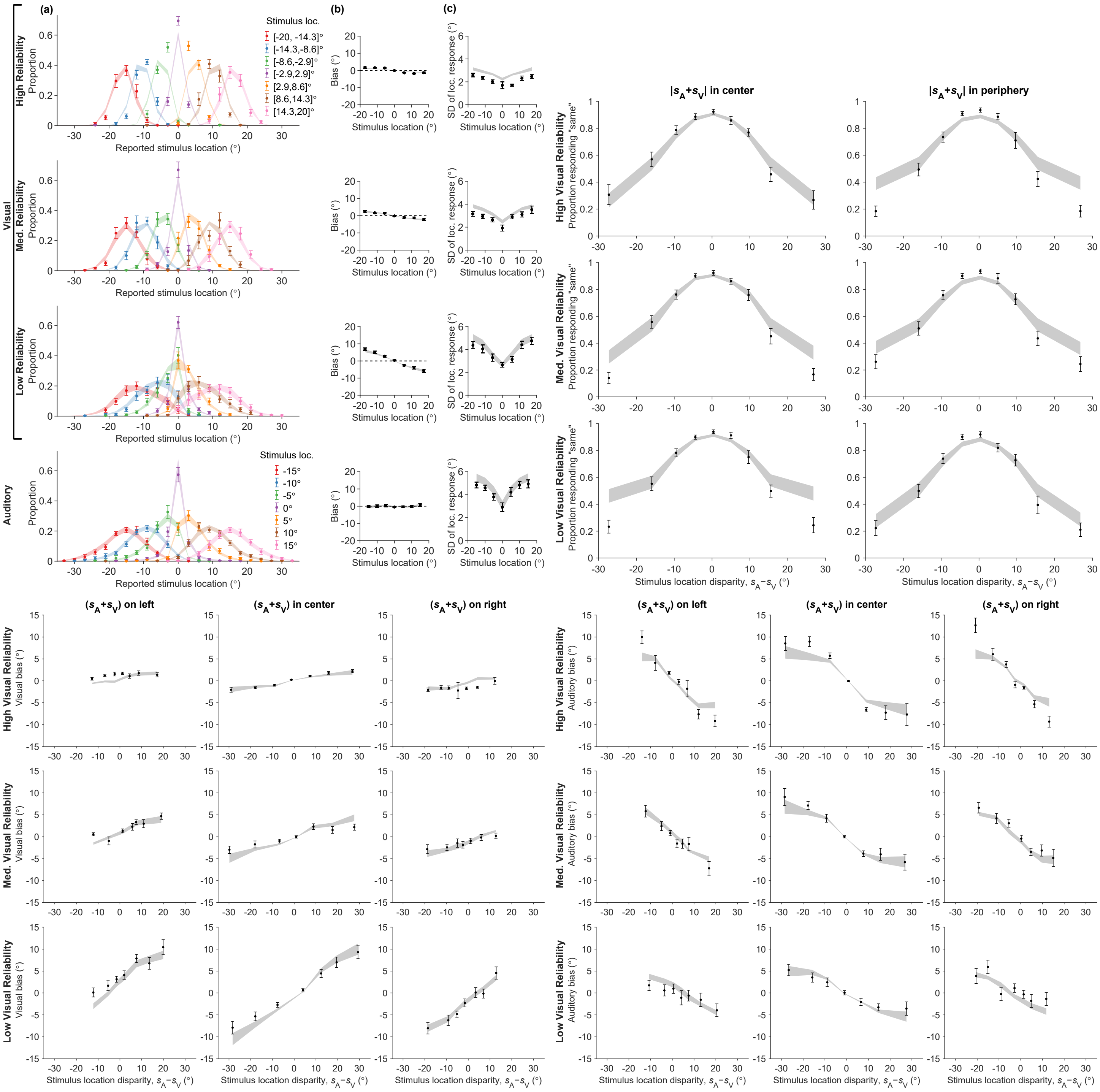

Fig L. The lifted-semiparametric fits on all tasks, assuming the PM causal inference strategy. Subplot notations are identical to Fig J.

Section C.3 All-tasks parametric model response distributions

We will only visualize the two best models fitted on all tasks.

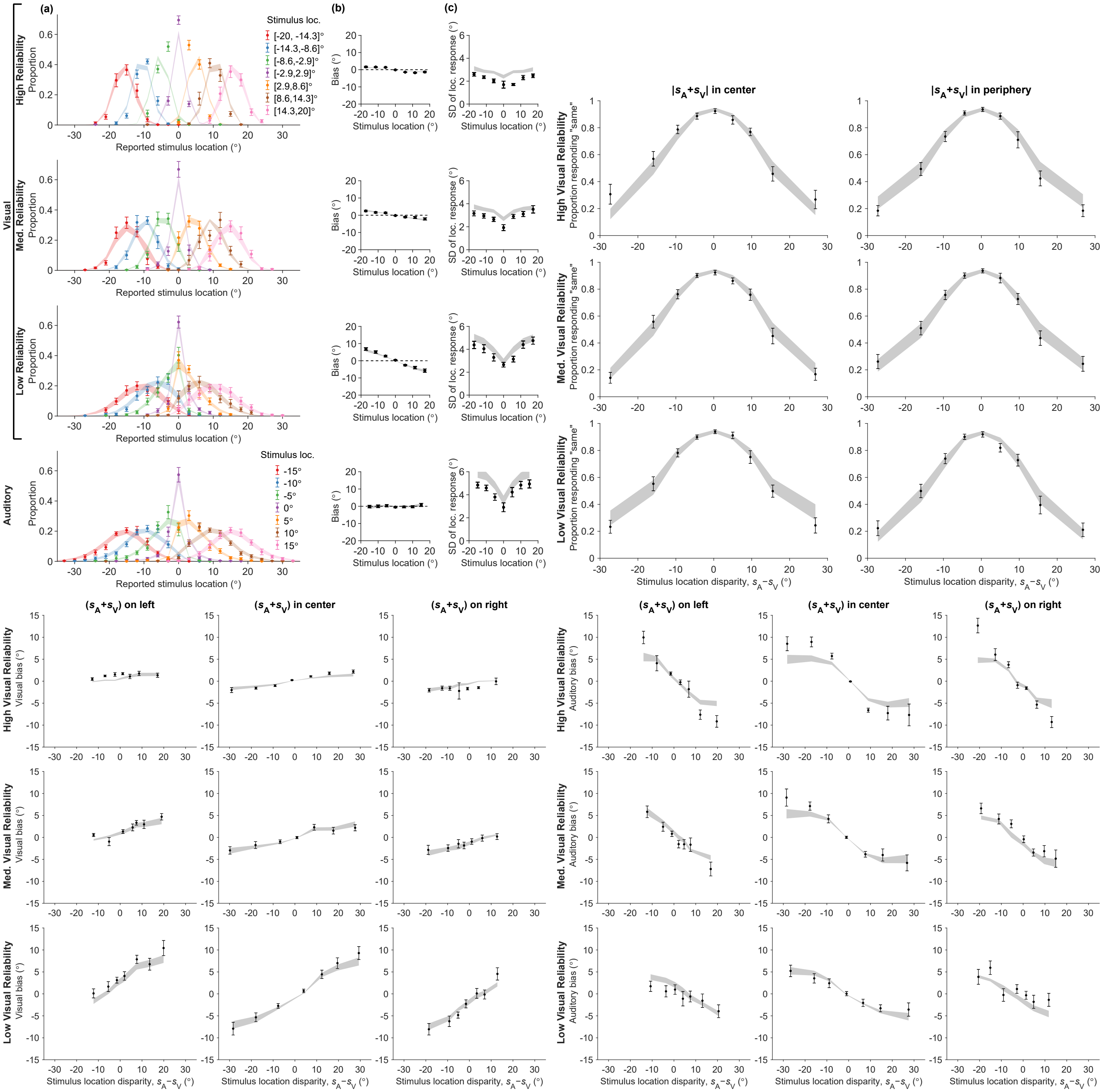

Fig M. The Exp-GaussianLaplace-MA parametric model fitted on all tasks. Subplot notations are identical to Fig J.

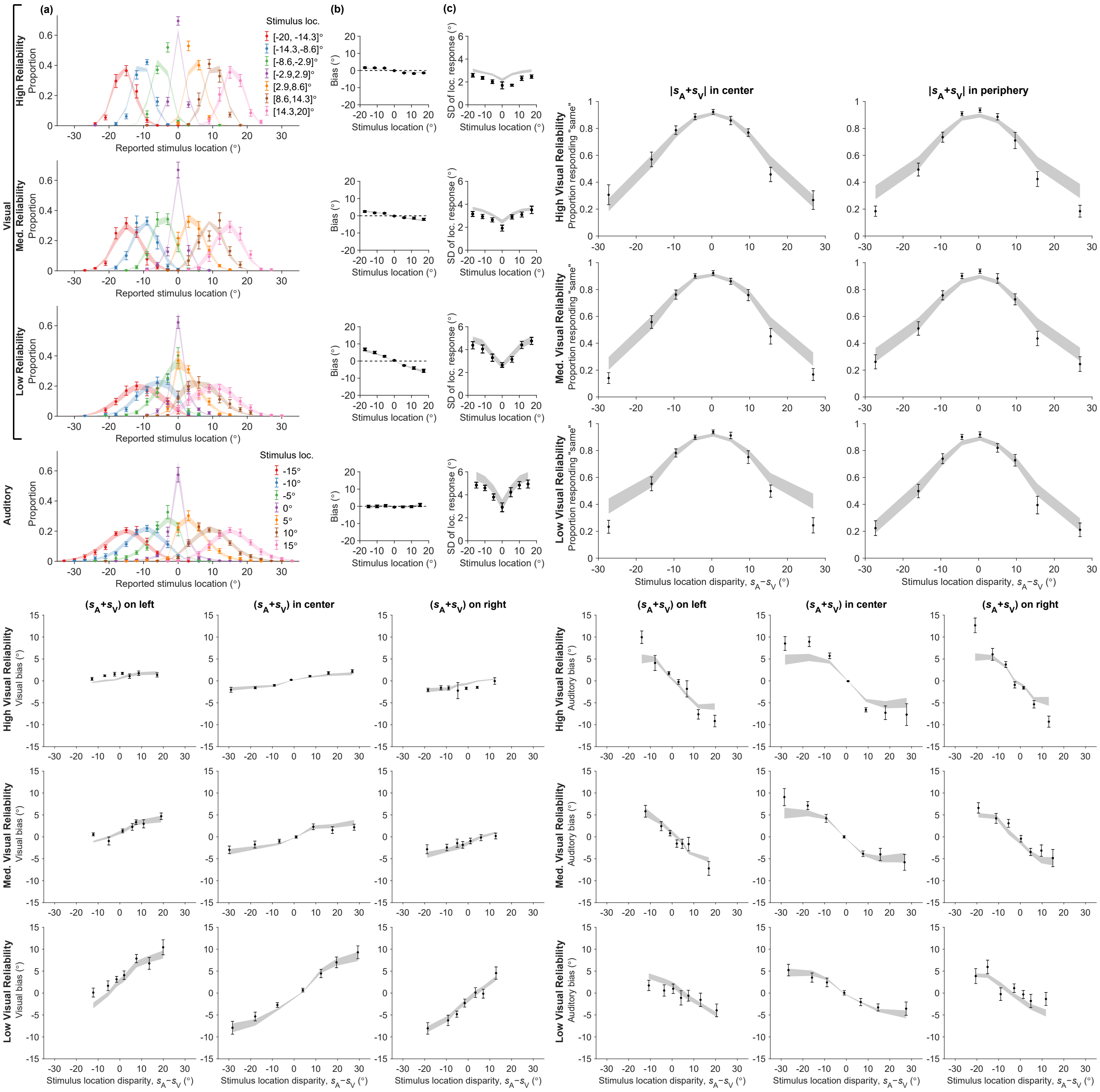

Fig N. The Exp-GaussianLaplace-PM parametric model fitted on all tasks. Subplot notations are identical to Fig J.

Section D Model response distributions, individual participants

We provide here individual-level model visualizations for the best parametric models fitted on either the unisensory data (Exp-GaussianLaplace) or all the data (Exp-GaussianLaplace-PM).

Section D.1 Unisensory data fit

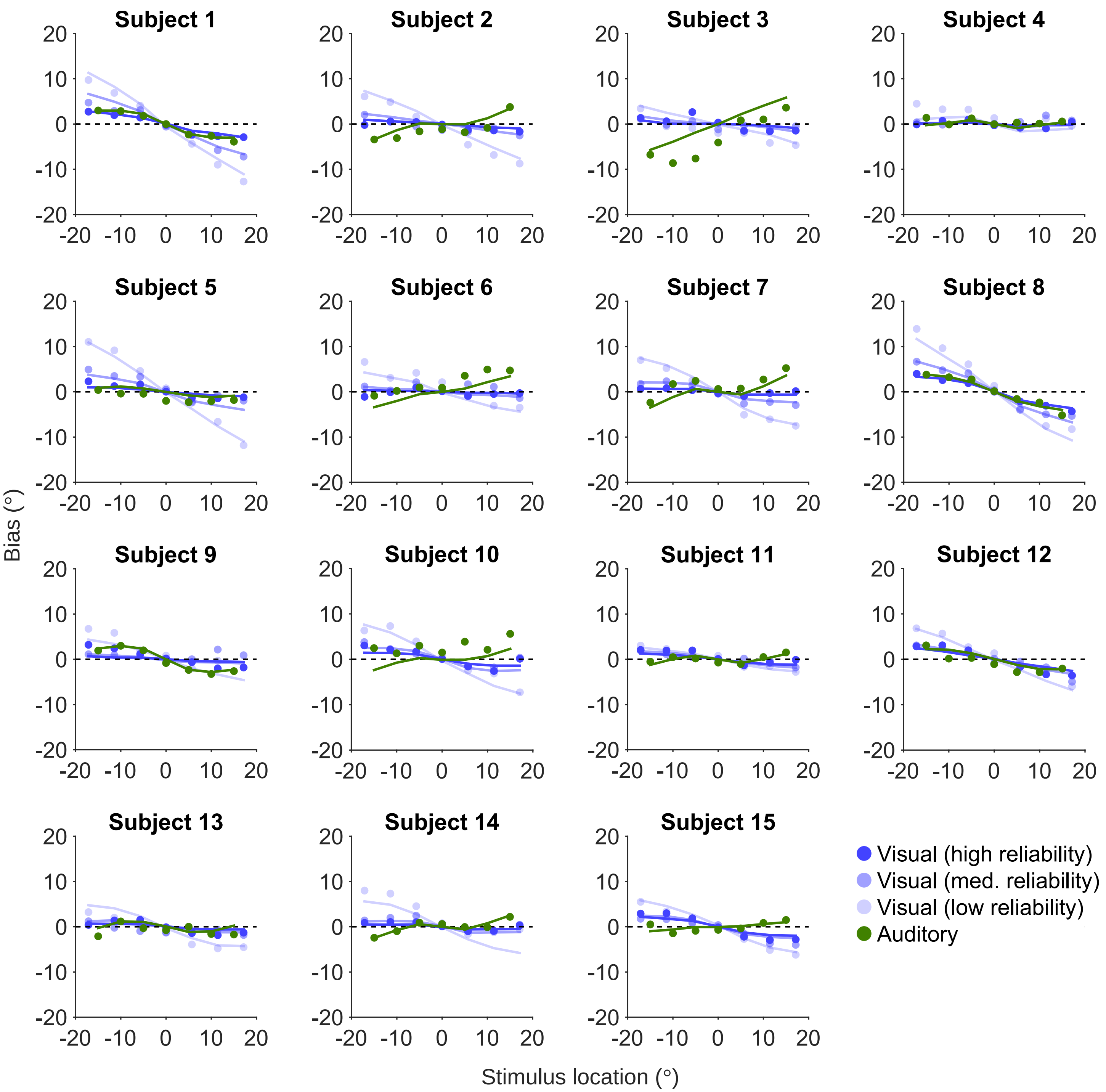

Fig O. Bias of UV and UA responses of each human participant and their Exp-GaussianLaplace parametric models fitted jointly on UV and UA data. Each participant is plotted in one subfigure. Different colors correspond to different visual reliability levels and sensory modalities. The trial-binning process is identical to Fig 2B in the main text, but with points denoting human data and lines denoting model predictions.

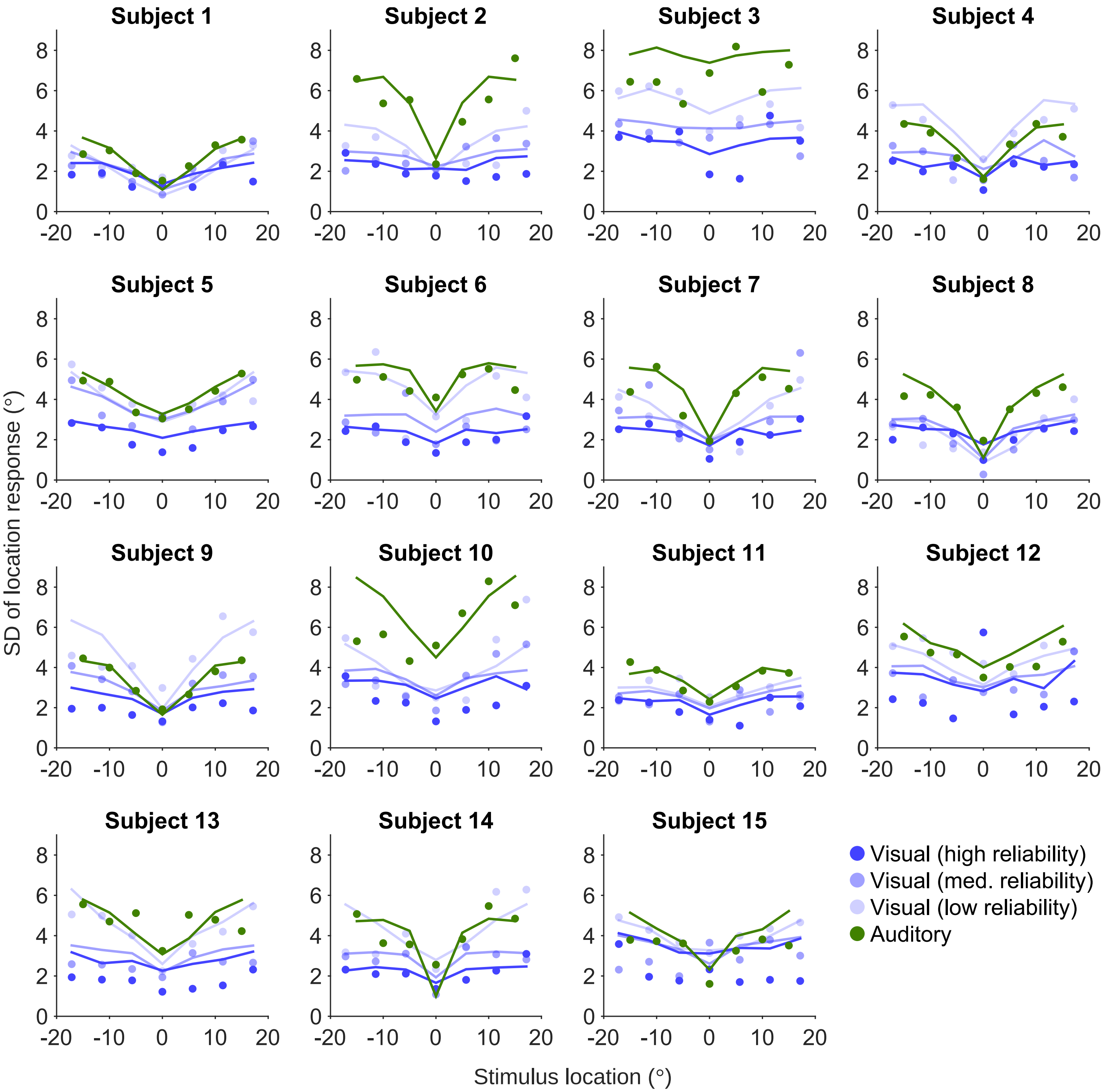

Fig P. SD of UV and UA responses of each human participant and their Exp-GaussianLaplace parametric models fitted jointly on UV and UA data. Each participant is plotted in one subfigure. Different colors correspond to different sensory modalities; different transparencies correspond to visual reliability levels. The trial-binning process is identical to Fig 2C in the main text, but with points denoting human data and lines denoting model predictions.

Section D.2 All-tasks fit

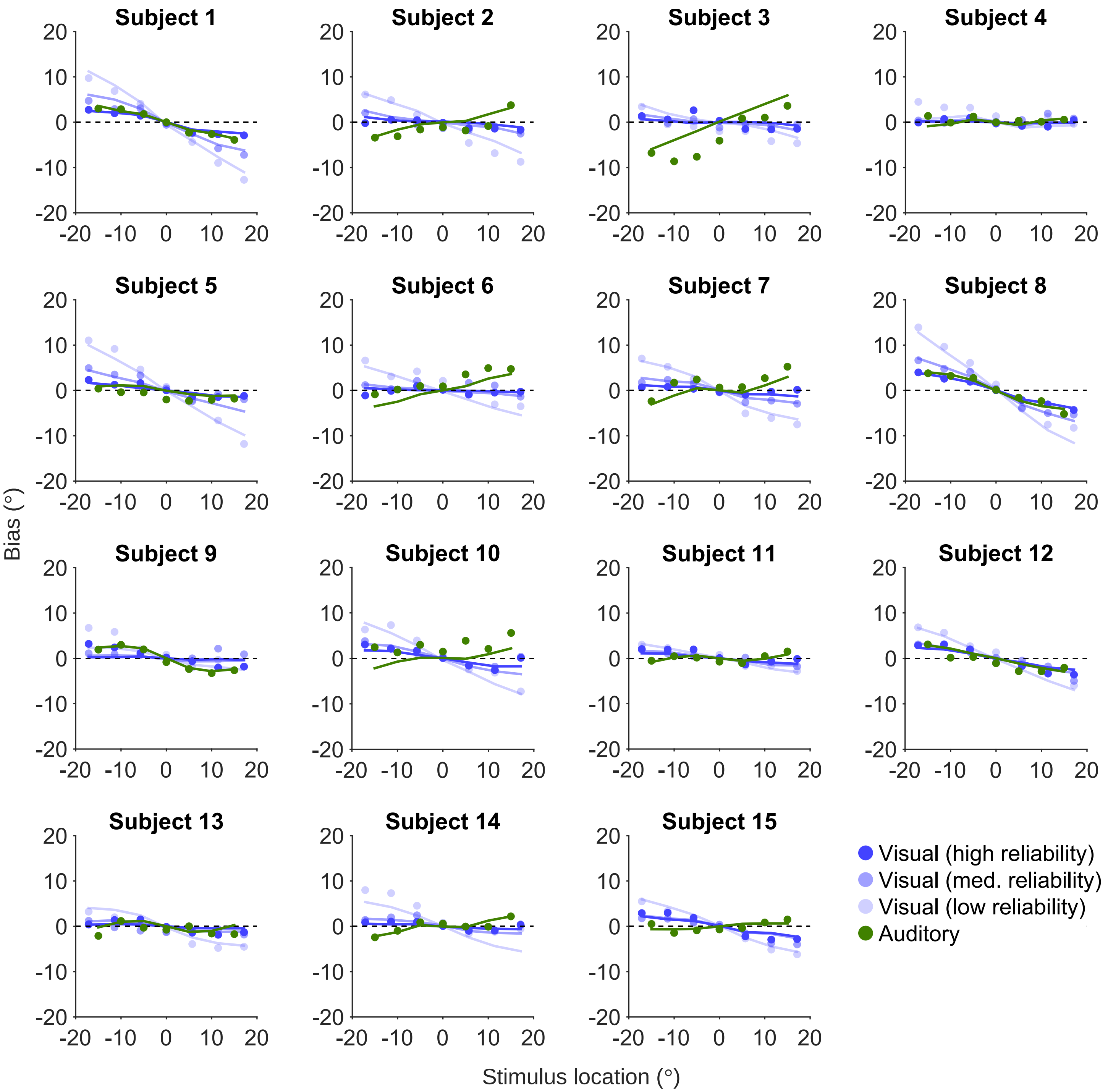

Fig Q. Mean UV and UA responses of each human participant and their Exp-GaussianLaplace-PM parametric models fitted on all tasks. The visualization process is identical to Fig O.

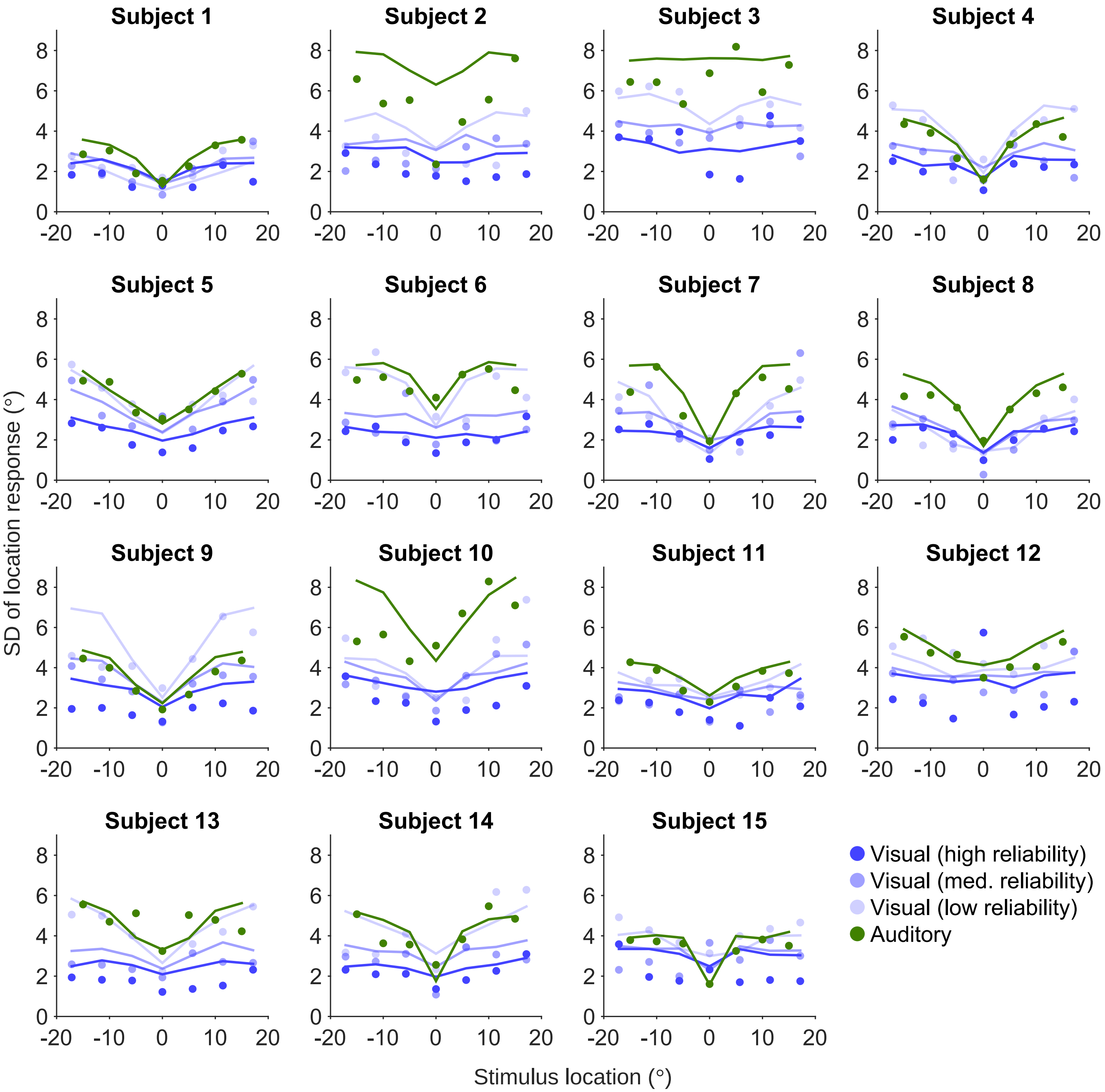

Fig R. SD of UV and UA responses of each human participant and their Exp-GaussianLaplace-PM parametric models fitted on all tasks. The visualization process is identical to Fig P.

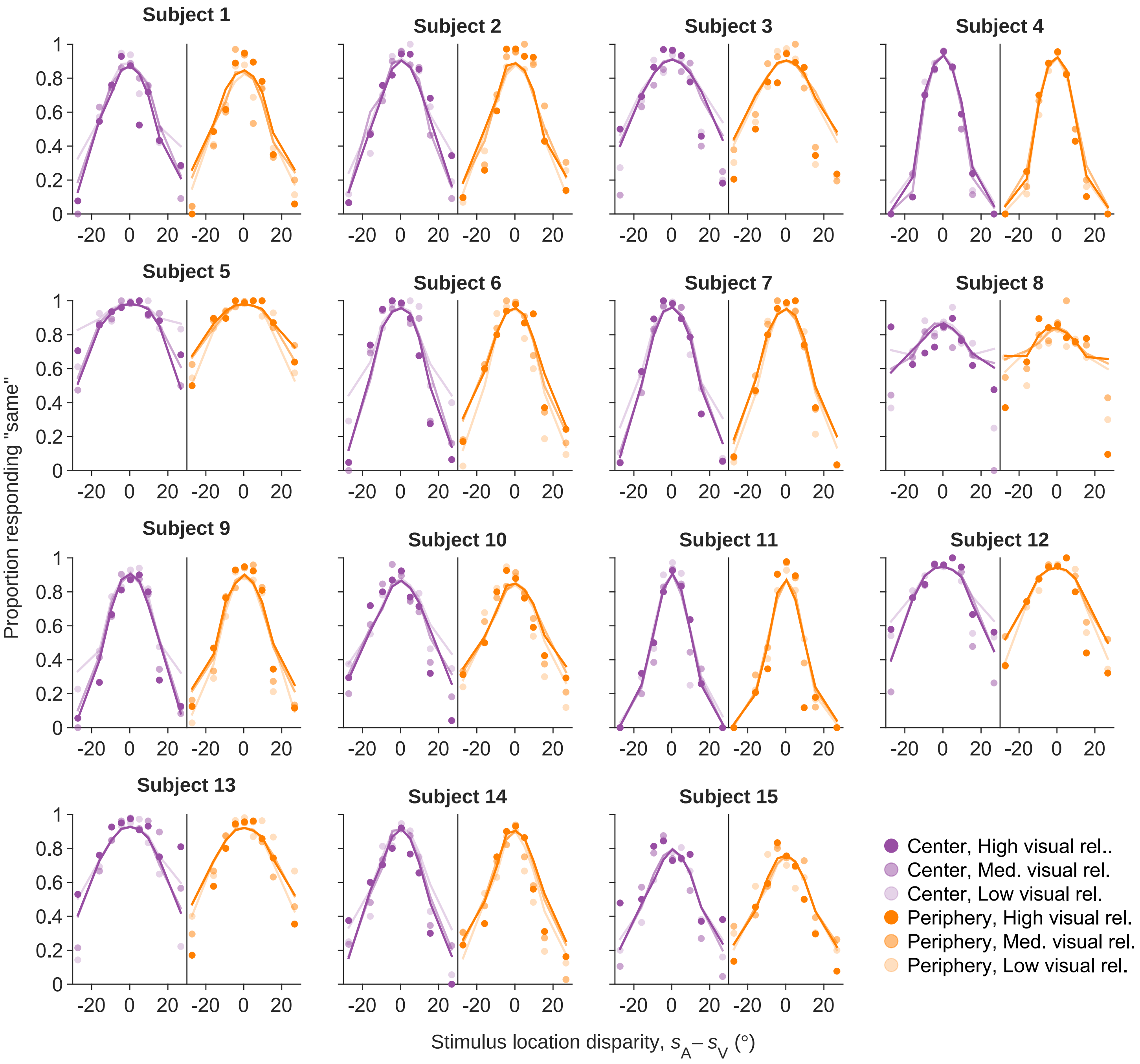

Fig S. BC responses of each human participant and their Exp-GaussianLaplace-PM parametric models fitted jointly on all tasks. Each participant is plotted in one subfigure. Different colors correspond to whether trial-specific $|s_A + s_V|$ is below (center) or above (periphery) its median value across all trials. The center-periphery-stratification and trial-binning processes are identical to Fig 6A in the main text, but now with points denoting human data and lines denoting model predictions.

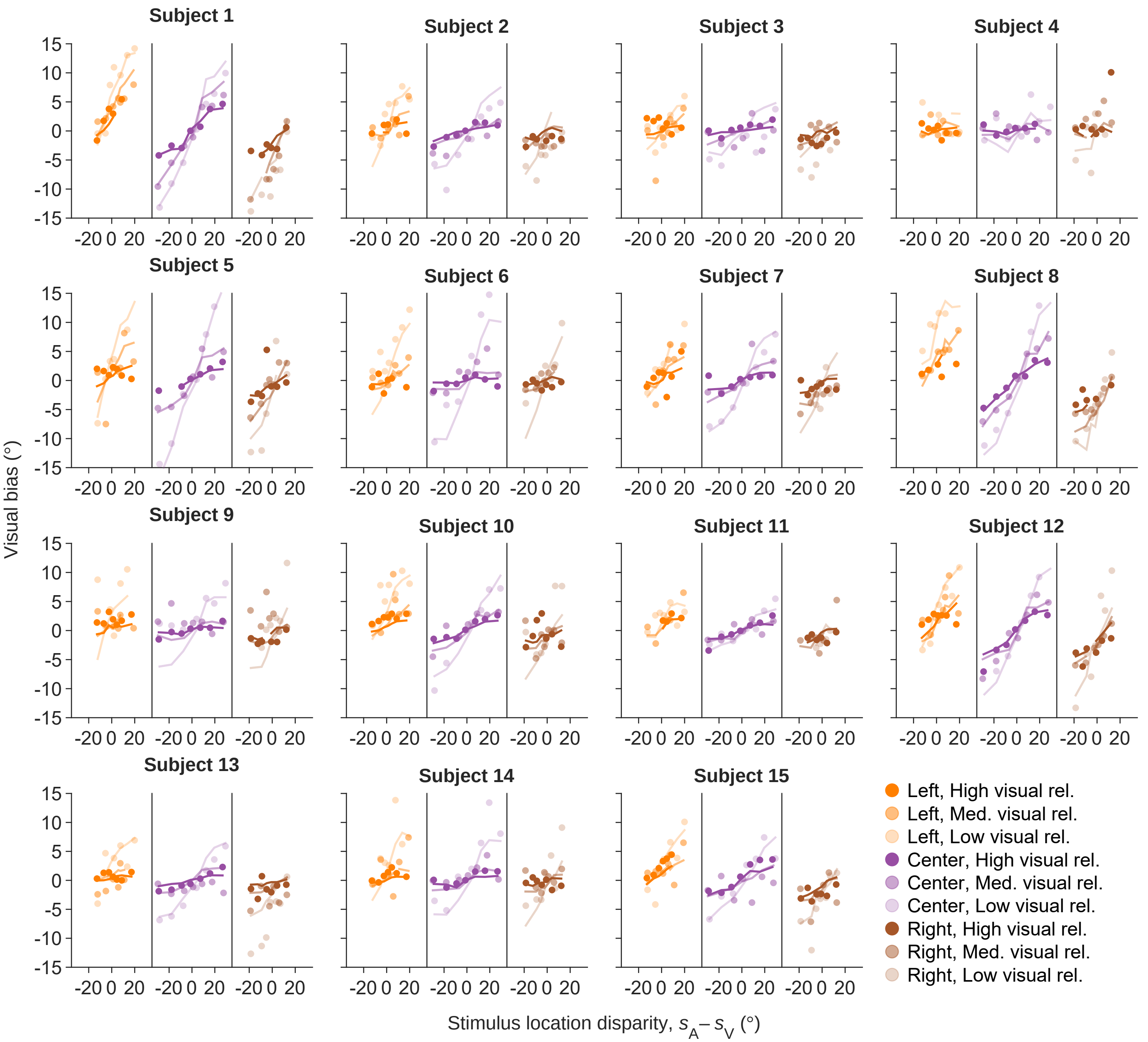

Fig T. BV responses of each human participant and their Exp-GaussianLaplace-PM parametric models fitted jointly on all tasks. Each participant is plotted in one subfigure. Different colors correspond to whether trial-specific $(s_A + s_V)$ is stratified into the left, center, or right group. The trial-stratification and trial-binning processes are identical to Fig 6B in the main text, but now with points denoting human data and lines denoting model predictions.

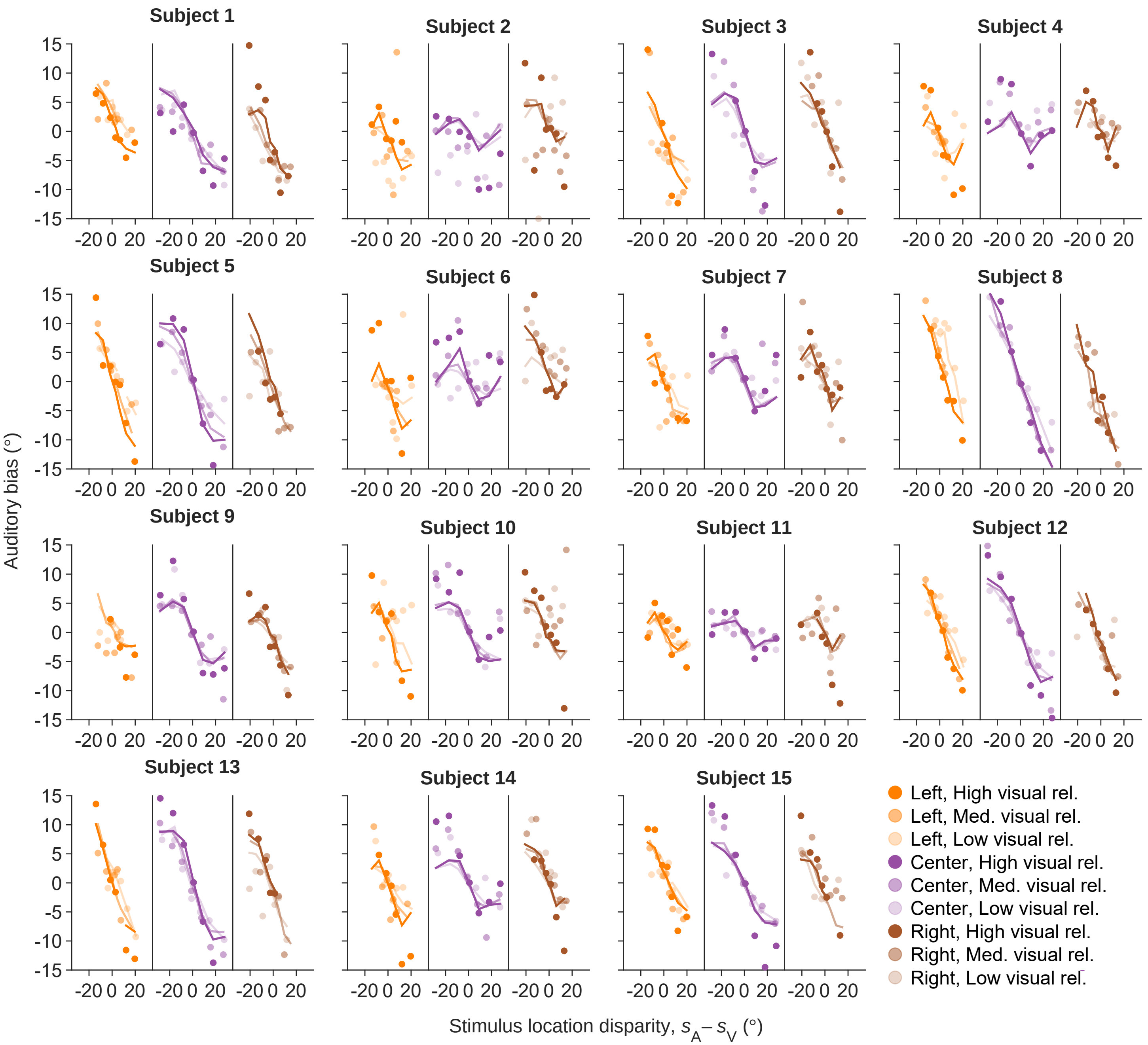

Fig U. BA responses of each human participant and their Exp-GaussianLaplace-PM parametric models fitted jointly on all tasks. Each participant is plotted in one subfigure. Different colors correspond to whether trial-specific $(s_A + s_V)$ is stratified into the left, center, or right group. The trial-stratification and trial-binning processes are identical to Fig 6B in the main text, but now with points denoting human data and lines denoting model predictions.

Section D.3 Individual fitted parameters

For each participant and the best-fitting Exp-GaussianLaplace-PM model (fitted over unisensory and bisensory data), we report their individual fitted parameters below. For other models, each participant's fitted parameters are available in the Github repository.

Table J. Individual fitted parameters for the Exp-GaussianLaplace-PM model.

Sub.	$\sigma_{0,\mathrm{V}}$	$k_{1,\mathrm{V}}$	$k_{2,\mathrm{V}}$	α_{med}	α_{low}	σ_s	λ	b	w	σ_{motor}	$\sigma_{0,\mathrm{A}}$	$k_{1,\mathrm{A}}$	$k_{2,\mathrm{A}}$	ρ_{A}	p_{same}	β_{V}	β_{A}
1	0.37	1.41	2.15	1.69	2.65	4.19	0.00	1.38	0.93	0.25	2.37	5.68	0.03	1.35	0.77	1.33	1.52
2	0.86	8.45	0.01	1.63	3.65	6.64	0.01	0.12	0.42	0.82	5.37	0.00	9.11	2.08	0.91	1.10	1.16
3	0.97	1.41	0.18	1.89	3.02	11.63	0.01	14.75	0.00	1.51	5.09	0.08	9.08	1.68	0.94	1.00	1.86
4	0.76	1.14	0.07	1.77	3.07	20.53	0.00	1.68	0.63	0.25	1.82	5.42	0.03	1.07	0.89	1.21	1.25
5	1.42	2.40	0.06	1.92	3.95	5.14	0.00	4.59	0.60	0.25	3.52	6.52	0.02	1.25	0.98	1.03	1.55
6	0.49	1.03	0.40	1.93	4.95	10.42	0.00	0.71	0.45	0.25	3.35	4.70	0.02	1.50	0.97	1.06	1.29
7	0.98	19.25	0.00	1.59	2.80	7.09	0.00	1.49	0.97	0.25	2.35	1.74	0.24	1.64	0.94	1.17	1.44
8	1.12	2.64	0.06	1.57	3.10	4.56	0.00	1.03	1.00	0.25	2.96	2.66	0.27	1.94	0.79	1.17	1.90
9	0.75	2.11	0.09	1.54	3.08	17.4	0.01	2.29	0.68	0.25	3.28	0.65	0.37	0.96	0.87	1.18	1.27
10	0.38	1.98	0.67	1.41	2.56	9.64	0.01	2.92	0.97	0.25	4.15	4.80	0.04	1.82	0.86	1.07	1.46
11	0.14	1.80	0.46	1.28	1.83	6.67	0.00	3.74	0.99	0.25	2.90	0.61	0.06	1.24	0.81	1.13	1.30
12	1.07	1.65	10.00	1.18	1.98	6.00	0.01	5.09	0.21	0.29	4.15	6.22	0.02	1.25	0.94	1.25	1.37
13	0.89	0.68	0.48	1.71	3.49	16.02	0.00	3.61	0.84	0.25	4.05	0.08	7.95	1.20	0.92	1.11	2.05
14	0.48	1.28	1.74	1.68	3.44	0.10	0.00	5.65	0.42	0.25	2.32	6.42	0.02	1.32	0.84	0.98	1.73
15	2.58	0.48	0.02	1.08	1.87	7.13	0.00	0.20	0.24	0.25	1.32	2.28	0.48	1.31	0.71	1.08	2.36
