# Supplementary material for: Distilling noise characteristics and prior expectations in multisensory causal inference
Source: PLoS Comput Biol. 2026 May 8;22(5):e1014251. doi: 10.1371/journal.pcbi.1014251 (PMC13155690; doi:10.1371/journal.pcbi.1014251)
Supplement: Attachment — Submitted filename: response_to_reviewers_r1.pdf [file pcbi.1014251.s003.pdf]

Dear Dr. Liu, Dr. van Vugt, and Reviewers,

Thank you very much for a constructive round of peer reviews. We have revised our manuscript according to each Reviewer's comments, and provide a detailed comment-by-comment reply below. Here, we also include a summary of the main revisions:

1. Introduction
   - Added discussion of efficient coding frameworks and their adaptations to naturalistic stimulus statistics (R2).
   - Expanded background on model comparison principles, including the roles of goodness-of-fit and parsimony in motivating AIC/BIC (R1).
2. Results
   - Clarified which behavioral patterns the *Constant-SingleGaussian* model failed to capture despite including the auditory recalibration parameter (R3).
   - Explained more explicitly how the sharply peaked location prior $p(s)$, heteroskedastic sensory noise $\sigma(s)$, and auditory recalibration parameter ρ_{A} jointly account for human behavior (R2, R3).
   - Noted that even the best-fitting model could not fully explain the strong central tendency observed under high visual reliability (R2).
3. Discussion
   - Addressed the alternative hypothesis of modality-specific location priors (R3).
   - Elaborated on how non-Gaussian environmental statistics might promote flexible prior adaptation and potential top-down adjustments in humans (R3).
   - Acknowledged limitations in not semiparametrically varying the causal inference strategy and highlighted heuristic alternatives proposed in recent work (R3).
4. Methods
   - Elaborated on participant instructions for each experiment condition, confirming that they were not given the precise probability of same-source trials (R3).
   - Added Table 1 summarizing all fitted candidate models and their degrees of freedom (R2).
   - Included model recovery and parameter recovery analyses (R3).
5. Supporting Information
   - Provided individual-level fitted parameters for the best-fitting Exp-GaussianLaplace-PM model across all data (R3).

Reviewer 1:

Overall summary

This study tests key assumptions commonly made in modeling multisensory integration within the Bayesian Causal Inference framework—namely, the assumptions of noise homoskedasticity and Gaussian priors. Drawing on a novel psychophysical experiment, the authors conclude that these assumptions are not supported by empirical data. They employ a semiparametric approach to infer the shape of the sensory noise and priors, leading to a model that fits the data more accurately than conventional approaches. This is an interesting and well-executed paper. Both the theoretical framing and experimental design are solid, and the methodological innovations are compelling. The manuscript is clearly written, and the data analyses are rigorous and thorough.

We thank the Reviewer for their positive feedback, and will address the remaining issues raised.

Major comments

The newly proposed model indeed achieves a better fit, but at the cost of introducing more free parameters. While the authors rightly justify this with model comparisons (e.g., via BIC), I would encourage a more explicit discussion of the trade-offs involved. Specifically, models with many free parameters, even if statistically favored, may sacrifice parsimony and interpretability. This raises important questions about falsifiability: with sufficient flexibility, nearly any dataset could be accommodated. This is a general challenge in the field, not a flaw of this particular model, but it would be valuable to foreground this issue for the benefit of readers.

We thank the Reviewer for raising this important point. We agree that statistical model comparison, while necessary, does not fully resolve concerns about parsimony and falsifiability—a model may win on BIC yet still be too flexible to constitute a meaningfully constrained theory.

We are acutely aware of this issue and have designed our modeling approach with it in mind. The ideal approach would be *pure prediction*: estimate all parameters in one condition and predict behavior in a different condition with no free parameters. Our approach does not fully achieve this—some auxiliary parameters are still fitted to the bisensory data—but it can be seen as a step in that direction. Specifically, we estimate the key model components (the sensory noise functions and prior shape) from unisensory data alone, then "lift" these estimates to the bisensory conditions, rather than refitting the entire model per condition. The lifted-semiparametric model must therefore generalize to a degree across task contexts, providing a stronger test than allowing all parameters to vary freely. Additionally, we "distill" parsimonious parametric model families from the shapes revealed by our flexible semiparametric fits—and notably, these simpler models outperform the semiparametric models on AIC/BIC precisely because the penalty for complexity outweighs the marginal improvement in raw fit. Finally, the parameters themselves correspond to psychophysically interpretable quantities, and in some cases their fitted values align with independent experimental constraints (e.g., the recalibration parameters approximating the ratio of stimulus ranges).

That said, the Reviewer is correct that this remains a broader challenge in the field, and we have added a discussion of these trade-offs in the Introduction:

> To provide a faithful account of human causal inference and bisensory perception, we adjudicate among candidate models through quantitative model comparison. The inclusion of additional model parameters (allowing for heteroskedastic sensory noise and non-Gaussian priors) would inevitably capture human behavior better than the baseline Bayesian model nested within. However, an unlimited expansion of model components is also undesirable, as it sacrifices interpretability and increases the risk of overfitting [36]. Following common practice in computational modeling, we utilize the Akaike and Bayesian Information Criteria (AIC/BIC) [39]. Both these model comparison methods penalize the number of free parameters on top of each model's goodness-of-fit, thereby striking a balance between simplicity and explanatory power [7]. That said, statistical model comparison alone does not fully address concerns about flexibility: a model may be statistically favored yet remain too unconstrained to constitute a meaningful theory. We therefore adopt two complementary strategies beyond AIC/BIC: first, we estimate key model components from unisensory data and "lift" them to bisensory conditions—requiring the model to generalize these components across task contexts rather than fitting each independently; second, we "distill" parsimonious parametric model families from the shapes revealed by flexible semiparametric fits.

The "distilled" parametric models are introduced as more tractable alternatives to the semiparametric approach. However, it would help to clarify how these distilled models should be treated: Are they intended as new normative models of perception, or simply as fitting tools? If they are meant to be candidate perceptual models in their own right, more discussion is warranted on whether their specific functional forms (e.g., exponential noise, Gaussian-Laplace priors) have perceptual or neurophysiological plausibility. Otherwise, the reader is left uncertain whether to interpret these models as descriptive conveniences or theoretical proposals.

We thank the Reviewer for this clarifying question. Our stance is that the distilled parametric models are candidate perceptual models, but our theoretical commitment is to their *qualitative features* rather than their exact functional forms. Specifically, we are committed to the claims that (1) sensory noise varies

with stimulus eccentricity, being lowest near center and higher in the periphery, and (2) the prior has structure beyond a single Gaussian, featuring a central peak that produces the observed concentration of responses at the midline. The specific functional forms (exponential noise, Gaussian-Laplace mixture) were chosen because they are simple, tractable, and sufficient to capture these features—not because we believe perception literally implements these functions.

This interpretation is supported by our finding that multiple parametric families (Exp-GaussianLaplace, Exp-TwoGaussians, Const-GaussianLaplace) perform comparably well. The data constrain the qualitative shape but do not uniquely determine the functional form, which is appropriate given current knowledge. We see this as a virtue: the distillation process identifies the minimal parametric structure needed to capture what the semiparametric fits revealed, without overfitting to incidental details.

Both qualitative features have independent support in the literature. Previous work in heading direction and spatial localization has shown that sensory variability varies with stimulus location, being lowest straight-ahead and higher in the periphery [8, 17, 28, 1, 40, 41, 26]. Non-Gaussian priors are motivated by efficient coding frameworks, in which stimulus representations are optimized for environmental statistics [38, 30, 31, 18]. We have clarified this interpretive stance in the Discussion of the revised manuscript:

> We emphasize that our theoretical commitment is to the qualitative features of these models—eccentricity-dependent sensory noise and a prior with structure beyond a single Gaussian—rather than their exact functional forms. The specific parametric shapes (exponential noise, Gaussian-Laplace mixture) were chosen for simplicity and tractability, not because we believe perception literally implements these functions. Indeed, multiple parametric families (Exp-GaussianLaplace, Exp-TwoGaussians, Const-GaussianLaplace) perform comparably, suggesting that the data constrain the qualitative shape without uniquely determining the functional form.

The findings offer convincing evidence for re-examining our modeling assumptions. That said, questions about generalizability remain. Would the new model outperform standard models on other datasets using similar paradigms? For example, two relevant studies with publicly available data are:
Noel, J. P., Shivkumar, S., Dokka, K., Haefner, R. M., & Angelaki, D. E. (2022). Aberrant causal inference and presence of a compensatory mechanism in autism spectrum disorder. *Elife*.
Mohl, J. T., Pearson, J. M., & Groh, J. M. (2020). Monkeys and humans implement causal inference to simultaneously localize auditory and visual stimuli. *Journal of Neurophysiology*.
Both of these studies use similar tasks but differ in key methodological aspects—particularly in how responses are collected. The current study relies on a visual pointer to report stimulus location. This may introduce asymmetries: in auditory-only trials, participants respond using visual feedback, whereas in visual and audiovisual trials, both stimulus and response are visually mediated. This difference could potentially bias modality weighting. Neither of the above studies face this issue, and showing that the new model generalizes to those datasets would significantly strengthen the present claims.
While the current model is evaluated using standard, low-level stimuli such as Gaussian blobs, it would be helpful for the authors to comment on how their modeling assumptions might adapt when dealing with more complex, naturalistic stimuli. In particular, real-world signals may not conform to Gaussian statistics, and it would be useful to discuss how the structure of priors and noise models might need to evolve under such conditions.

We thank the Reviewer for these thoughtful suggestions regarding generalizability.

Regarding testing on other datasets: we agree this would be valuable, and we appreciate the Reviewer pointing to specific publicly available datasets. However, applying our full modeling pipeline to new datasets—each with different paradigms, response modalities, and stimulus configurations—would constitute a substantial undertaking beyond the scope of the current paper. We have made our code openly available at `https://github.com/LSZ2001/Audiovisual-causal-inference` precisely to enable such cross-dataset comparisons by ourselves and others in future work.

Regarding the visual pointer method: the Reviewer raises a valid concern. We acknowledge that using a visual response modality across all conditions could in principle influence behavior, and, as mentioned in the paper, we suspect it may contribute to the auditory range recalibration effect we observe. However, we are less certain that it would affect our core findings regarding heteroskedastic sensory noise and non-Gaussian

priors, which are estimated primarily from the structure of (unisensory) response distributions rather than from asymmetries across modalities. That said, we agree this is worth investigating, and datasets using different response methods (such as those the Reviewer mentions) would provide a useful test. We have added a note on this limitation in the Discussion:

> Additionally, our use of a visual pointer for all responses means that auditory-only trials required cross-modal responding, which could in principle influence behavior; we suspect this may contribute to the recalibration effect, though this is unlikely to affect our core findings, which derive from the structure of unisensory response distributions rather than cross-modal comparisons.

Finally, regarding naturalistic stimuli: the qualitative features we identify—eccentricity-dependent noise and structured priors—may well apply beyond laboratory settings, though the specific functional forms would likely need reassessment given the richer statistics of natural environments. We touch on this briefly in the Discussion.

Finally, a recent study (Parise, 2025, eLife) introduces an algorithmic, stimulus-computable implementation of the Bayesian Causal Inference framework. This model operates with a small number of free parameters and does not rely on priors. It would be valuable for the authors to discuss how their approach compares to this algorithmic model—particularly in terms of theoretical commitments, flexibility, and the trade-off between parameter-rich fitting and process-level explanation.
Parise, C.V. (2025). A Stimulus-Computable Model for Audiovisual Perception and Spatial Orienting in Mammals. *Elife.*

We thank the Reviewer for highlighting this recent work. Parise (2025) proposes that audiovisual integration and causal inference can emerge from correlation detection between sensory streams, operating directly on stimulus input with few free parameters. This represents a different level of analysis from our approach: whereas we aim to characterize *what* statistical assumptions (priors, noise structures) best describe human behavior at Marr's computational level, Parise addresses *how* such inferences might be implemented through biologically plausible mechanisms.

We see the approaches as complementary rather than competing. Correlation-based mechanisms implicitly embody assumptions about the statistical structure of the environment—namely, that correlated signals tend to originate from common sources. Our work asks what the functional form of those assumptions must be to account for human behavior; process-level models like Parise's offer candidate mechanisms that could give rise to them. We have added this paragraph to the Discussion:

> Recent work has proposed process-level models for audiovisual integration based on correlation detection between sensory streams, which can account for causal inference phenomena with few free parameters [29]. Such algorithmic approaches address a complementary question to ours: whereas we characterize what statistical structure best describes human behavior, process-level models address how such inferences might be implemented. Bridging these levels of analysis—by identifying what architectural features of process-level models give rise to behavioral signatures like central response biases and eccentricity-dependent variability—represents a promising direction for future work.

Reviewer 2:

Overall summary

In this manuscript by Shuze Liu et al, the authors proposed and tested a set of Bayesian observer models for visual-auditory causal inference tasks. The key idea is to development more general models that relax the assumptions of homoskedastic sensory noise and Gaussian priors. The authors proposed several variations of models, and used these models to study several unisensory and bisensory tasks. Notably, the model parameters inferred in the unisensory tasks were used to inform the model for bisensory tasks. The authors also distilled a new parametric model inspired by the results on the more complex semi-parametric models.

Overall, this is an impressive and well executed study. I found the overall results to be interesting and worthwhile. The authors proposed and empirically tested several versions of Bayesian models using behavioral data from multiple experimental tasks. The analyses and model comparisons are systematic and thorough.

I have a few modest to substantial concerns. I hope these could be addressed in a revision.

We appreciate the Reviewer's feedback and will address the concerns raised.

Major comments

1. My first concern is about the overall framing of the study. The main innovation of this paper is to include more flexible sensory noise and priors to the model for modeling the visual-auditory causal inference tasks. While classic Bayesian models often make simplified assumptions of noise and priors, models with more flexible sensory noise and priors have also been proposed and tested over the past decade (e.g., Wei & Stocker, *Nature Neuroscience*, 2015; Polania, Woodford & Ruff, *Nature Neuroscience*, 2019; Prat-Carrabin & Woodford, *Nature Human Behaviour*, 2022; Hahn & Wei, *Nature Neuroscience*, 2024). In particular, Hahn & Wei (2024) described a flexible Bayesian model fitting framework that takes into account arbitrary smooth priors and noise variability. These models focused on the study of unisensory task, in particular one-d estimation tasks.

I don't feel a direct comparison to the modeling procedures in Hahn & Wei (2024) for the unisensory cases is needed, but the paper would be stronger if the authors could engage and discuss that literature from the beginning of this paper. This would help clarify the main contributions of this paper.

Having said that, I would like to emphasize that the main models proposed for the bisensory tasks in this paper are certainty novel, and go beyond what was done previously. In particular, for these tasks, additional assumptions on the causal inference strategies need to be assumed. They authors tested three strategies, model averaging, model selection and probability matching.

We thank the Reviewer for the opportunity to clarify the relationship to prior work. We agree that flexible priors and noise have been explored in unisensory settings, particularly within efficient coding frameworks [38, 30, 31, 18]. Our contribution is to extend flexible inference of priors and sensory noise to the multisensory causal inference setting, where these components interact with causal inference strategies (model averaging, model selection, probability matching) to produce more complex behavioral patterns. We have revised the Introduction to better situate our work:

> Recent work has developed flexible approaches to inferring priors and sensory noise from behavior, particularly within efficient coding frameworks that link these components normatively to environmental statistics [38, 30, 31, 18]. However, these studies have focused primarily on unisensory estimation tasks. In the multisensory setting, priors and sensory noise interact with an additional layer of inference—the causal structure linking sensory signals—which introduces new complexities not addressed by existing frameworks. Our work extends flexible, data-driven inference of priors and noise to this richer setting.

2. In the semi-parametric model and the Exp-GaussianLaplace parametric model, what accounts for the near zero biases for the auditory task? It was not clear to me whether they are accounted for by the more flexible prior and noise, or by range recalibration discussed in earlier part of the paper.

We thank the Reviewer for identifying this source of confusion—the near-zero biases in the auditory task are captured by the auditory range recalibration parameter. Now when discussing the semiparametric fits, we clarify the contributions of each model component:

> To validate that the semiparametric fits explain our participants' data well... Overall, there is a good match between the model-predicted response distributions and the human response distributions. Importantly, the combination of a sharply peaked $p(s)$, heteroskedastic sensory noise $\sigma(s)$, and auditory range recalibration ρ_A has allowed the model to capture the behavioral signatures we observed in this task—the prior and sensory noise function induce strong central tendencies in UV, while auditory range recalibration maintains unbiased UA estimates amid the prior.

One more point about the range recalibration: This looks a bit post-hoc. I wonder if the authors have any other additional evidence for it beyond that including this mechanism can better explain the biases.

The Reviewer is absolutely correct in that the recalibration parameter was not anticipated in advance in our modelling endeavor. However, we believe there is evidence beyond improved fit: the fitted values cluster around 4/3, which corresponds to the ratio of visual to auditory stimulus ranges in our experiment. This correspondence would be difficult to explain if the parameter were merely absorbing noise in the data.

We now elaborate on related limitations in the Discussion:

> Notably, although the evidence remains circumstantial, the fitted recalibration parameters being approximately 4/3—which corresponds to the ratio of visual to auditory stimulus ranges in our experiment—lends support to our interpretation, as this correspondence would otherwise be difficult to explain.
>
> [...]
>
> Second, we acknowledge that the auditory range recalibration effect—where participants appeared to stretch the auditory stimulus range to match the visual range—may be specific to our experimental setup with mismatching visual and auditory ranges. [...] Still, our findings hint more generally at an interplay between sensory recalibration and multisensory causal inference which deserves further investigation [20].

Beyond our empirical results (model comparison), sensory recalibration has been proposed and observed in many past studies. These works have been cited in the manuscript:

> We introduced this parameter motivated by the well-known phenomenon of sensory recalibration in the presence of a consistent mismatch between modalities [15, 32, 33, 20, 28, 37, 12, 13, 10, 4], and its importance will be discussed and validated empirically in the next section.

3. For the high visual reliability condition, the fits are consistent off for the [-8.6,-2.9] deg and [2.9, 8.6] deg conditions, in particular around the peak. Can the authors comment on this?

We appreciate the Reviewer's close reading of the figures. We now comment on this in the Discussion:

> The large mass of the Gaussian-Laplace mixture prior in central angles is consistent with past literature [9, 8], reflecting particularly strong human expectations for stimuli to appear in the center-front. This prior expectation appears even stronger than what the current models could capture, as the Semiparametric and Gaussian-Laplace models were unable to reproduce the strong UV central tendencies in the high visual reliablity condition, when the stimulus location is in $[-8.6, 2.9]^\circ$ or $[2.9, 8.6]^\circ$ (Figs 5, 8, 14).

4. Some of the materials in Results may be merged into Methods to make the text flow better.

We thank the Reviewer for this suggestion. We agree that a more concise presentation would improve readability. However, much of the material currently in the Results is directly tied to our core contribution—the combinatorial model comparison process. Because we frequently refer to different model variants and their structural distinctions when interpreting the findings, we felt compelled to introduce these model components before presenting the behavioral results. We would be happy to consider specific examples that the Reviewer would suggest moving to the Methods section.

Minor comments

Line 377-379, the authors wrote "the fits still present the same general issues highlighted previously ". It may be useful to briefly mentioned the issues again here.

We apologize for the confusion here, and have updated the excerpt to read: "the fits still present the same general issues highlighted previously—failing to account for the strong central tendency and central SD dip as seen in humans".

In Fig. 9, the LiftedSemiparam-PM model contains two set of numbers. This is slightly confusing. It might be better to split that into two separate rows.

We have considered this alternative visualization strategy, but eventually decided to place both numbers in the same row because they correspond to the same fitted model. In the figure captions, we have provided relevant information:

> For the LiftedSemiparam-PM model, many of its parameters were fitted in the earlier semi-parametric fits. Its BIC score including such parameters is shown in white, and the BIC score excluding them is shown in gray.

The authors reported results based on a number of models and experimental conditions. While this is impressive, it also makes the paper more difficult to follow. I wonder if it would be useful to have a table to summarize these.

We thank the Reviewer for suggesting this addition, which has drastically improved the manuscript's readability. Aside from Figs 1 and 3 that depict the experimental conditions and modeling components, we have added Table 1 in the Methods section that documents the models fitted:

Table 1: Summary of models fitted. Numbers in parentheses convey the number of free parameters incurred by each modeling component (for sensory noise, the number of free parameters is multiplied by 2 due to separate $\boldsymbol{\theta}_{\mathrm{V}}, \boldsymbol{\theta}_{\mathrm{A}}$). The auditory range recalibration parameter is only systematically varied in the unisensory fits (*), and always free in bisensory fits. The semiparametric model is fitted only on unisensory data (**), while its fitted sensory noise and prior functions are then used for LiftedSemiparam models fitted to bisensory data (***). The acronyms MS, MA, and PM stand for model selection, model averaging, and probability matching causal inference strategies, respectively.

**Model**	**Sensory noise**	**Prior**	**Aud. recalib. (unisensory*)**	**Causal inf. strategy (bisensory)**
Exp-GaussianLaplace	Exponential (3×2)	Gaussian-Laplace mixture (3)	1, 4/3, free (1)	MS, MA, PM
Exp-SingleGaussian	Exponential (3×2)	Gaussian (1)	free (1)	MS, MA, PM
Const-GaussianLaplace	Constant (1×2)	Gaussian-Laplace mixture (3)	free (1)	MS, MA, PM
Exp-TwoGaussians	Exponential (3×2)	Two-Gaussian mixture (3)	free (1)	MS, MA, PM
Const-SingleGaussian	Constant (1×2)	Gaussian (1)	1, 4/3, free (1)	MS, MA, PM
Semiparametric** LiftedSemiparam***	Flexible (12×2)	Flexible (11)	free (1)	MS, MA, PM

Reviewer 3:

Overall summary

Liu and colleagues report an approach to estimating location-dependent sensory noise distributions and individual priors of unrestricted form. They estimate and then approximate these distributions in the context of Bayesian causal inference models fit to visual, auditory, and visual-auditory localization responses. The unusually skewed localization responses are best described by causal inference models with strongly heteroscedastic noise distributions and priors with the majority of mass in the center of the stimulus space. The approach is interesting and relevant beyond multisensory causal inference. However, I have concerns about a few untested model assumptions, the recoverability of the two approximated distributions, and am not convinced that the rather strong claims throughout the manuscript are warranted.

We appreciate the Reviewer's candid feedback, and we address each concern below.

Major comments

The unisensory visual localization data are fascinating, very consistently skewed towards the center across different levels of visual reliability. Visual and auditory localization response variability clearly varies with distance from the center. Both characteristics evidently drive the recovered noise functions and priors. However, I could not find any publication with indications of similarly extreme heteroscedasticity (and checks of our own data routinely reveal homoscedasticity in the auditory domain) or central tendencies in uni- or multisensory visual or auditory localization. Thus, these data might well be specific to the stimuli or testing protocol. This implies that 1) the conclusion that all previous models are wrong by assuming homoscedasticity or Gaussian priors seems not warranted, and 2) it would be good to account for the possibility that the effects are to some degree driven by response rather than sensory processes.

We thank the Reviewer for highlighting the general possibility that certain results were specific to our task protocol. We will address the pertinent comments point-by-point below.

Lapses are modeled using a uniform distribution, which makes sense for accidental mouse clicks if the cursor initially appears at random locations. However, a remark in the discussion suggests this was not the case, and that accidental clicks would tend to produce central responses. Additionally, since the stimuli are very brief, participants might miss them—perhaps because they blinked. For such lapses, smart participants would select locations in the center of the screen. Therefore, everything points towards a non-uniform lapse distribution with a sharp peak at the center.

We agree with the Reviewer that a centered bias distribution may be plausible—on every trial, the cursor was initialized to its response location on the previous trial, and participants do demonstrate a central tendency in their responses.

However, as described in the Appendix, we have fitted models with a Gaussian lapse distribution that peaks in the center, and found that it did not improve model fits:

> We have also considered a truncated-Gaussian lapse distribution with the response range $[-45, 45]$ to mitigate the problem. In contrast to the uniform lapse distribution $U[-45, 45]$ used in all previous models, the truncated-Gaussian lapse distribution requires the fitting of not only a lapse rate λ, but also the lapse distribution's standard deviation σ_{lapse}.
>
> We have fitted an Exp-GaussianLaplace model assuming Gaussian lapse to the unisensory data. Its visualization in Fig S2 reveals that using a truncated-Gaussian lapse distribution does not seem to resolve the SD overestimation problem. Furthermore, BIC model comparison results cannot distinguish the truncated-Gaussian lapse model from its uniform lapse counterpart featuring one less parameter. Specifically, the Sum and [2.5%, 97.5%] bootstrapping intervals for the BIC difference is -41.8 and [-114, 18.6] respectively, where the difference is computed with respect to the Uniform lapse model. For this reason, we used the standard uniform lapse model in the main text, leaving further investigations for future work.

Response noise is assumed to be independent of location. However, if participants really maintain fixation, the perception of the visual cursor should be noisier in peripheral stimulus regions. (If they moved their eyes, there's no reason to assume visual heteroscedasticity.) Moreover, if the cursor always appeared in the center, perceptual learning might quickly create an asymmetry in sensory precision across the visual field. Therefore, it would be good to test model variants with location-dependent response noise.

We thank the Reviewer for bringing this up. First, we hope to clarify that the cursor does not always appear in the center at the beginning of every trial:

> The cursor position was not reset to zero in each trial, preventing the formation of a central tendency effect just out of "laziness" in moving the cursor away from zero [2].

While studying location-dependent response noise would be interesting on its own (likely involving iterative cycles of cursor perception, path integration, and motor control), it lies orthogonal to the manuscript's main focus on characterizing human sensory noise, priors, and causal inference strategies during external stimuli perception. Specifically, location-dependent response noise would primarily affect response variability, but the central tendency we observe is a bias in mean responses, which is naturally explained by a peaked prior and/or heteroskedastic sensory noise rather than response noise alone. Furthermore, the current Exp-GaussianLaplace-PM model could already capture most aspects of human behavior, and hence believe it may be better suited for a follow-up project.

The authors' models are compared to "vanilla" models of Bayesian unisensory perception and Bayesian cross-modal causal inference. There is clearly value in fitting the simplest possible model to demonstrate its limitations in capturing the data's characteristics. However, the comparison to a vanilla model does not clarify whether the heteroscedasticity of sensory noise or the peaky prior causes the qualitative change in predictions. The AIC values indicate that the former is the case; however, it would be helpful to illustrate (and discuss) the role of each factor in replicating the observed data. Furthermore, it should be clarified that the vanilla models are straw men and not representative of the field.

Both heteroskedastic sensory noise and a peaked prior contribute to the central tendency in UV data. This conclusion is supported by AIC/BIC model comparisons, which prefer the Exp-GaussianLaplace model over Const-GaussianLaplace and Exp-SingleGaussian. Now when discussing the Semiparametric model fits, we confirm how each model component contributes to goodness-of-fit:

> Overall, there is a good match between the model-predicted response distributions and the human response distributions. Importantly, the combination of a sharply peaked $p(s)$, heteroskedastic sensory noise $\sigma(s)$, and auditory range recalibration ρ_{A} has allowed the model to capture the behavioral signatures we observed in this task—the prior and sensory noise function induce strong central tendencies in UV, while auditory range recalibration maintains unbiased UA estimates amid the prior.

Regarding whether the vanilla model is representative of the field, we have brought up lines of literature suggesting that it does constitute the default modeling choice across many studies:

> This is a baseline, standard Bayesian observer model commonly used in perceptual estimation [24, 34, 41, 27, 26, 6].

That said, we agree that these models are often adopted for tractability rather than out of genuine commitment to their assumptions. Our point is not that the field believes in homoskedastic noise and Gaussian priors, but that these assumptions have rarely been systematically tested or replaced with empirically grounded alternatives—which is what we aim to provide.

The tested models assume the existence of supramodal priors. However, this assumption is not supported by unimodal data and does not match the statistics of the natural world: objects we see clearly tend to be in the center of our vision because of poor resolution in the periphery, while many sounds we hear come from outside our visual field. The experimental stimulus distribution is supramodal (except for a difference in range) but very different from the recovered priors.

We are grateful to the Reviewer for bring up this point. We now discuss this alternative hypothesis in relation to our empirical data in the Discussion section:

> For unisensory conditions, the recalibration effect could alternatively be explained by assuming different location priors for vision and audition, with the auditory prior being relatively uninformative and thus producing minimal empirical bias in posterior location estimates. However, extending this modality-specific-prior account to bisensory trials would require a nontrivial modification of the model class, because both visual and auditory inputs may originate from a common source—which necessitates a shared prior $p(s)$ for evaluating the posterior probability of the $C = 1$ hypothesis. In other words, relaxing the supramodal prior assumption would require an entirely new class of models for Bayesian causal inference, which represents an interesting direction for future work. Notably, although the evidence remains circumstantial, the fitted recalibration parameters being approximately 4/3—which corresponds to the ratio of visual to auditory stimulus ranges in our experiment—lends support to our interpretation, as this correspondence would otherwise be difficult to explain. In line with past work [15, 32, 33, 20, 28, 37, 12, 13, 10, 4], we propose that the recalibration mechanism may extend to multisensory causal inference and encourage future research to test its generality.

I could not find any mention of model or parameter recovery. Both checks are relevant given the high dimensionality of the models. Here, it seems especially important to show that potential trade-offs between the recovered prior, likelihood/noise distribution, and lapse and response noise distributions are under control.

We thank the Reviewer for suggesting this addition. We have now performed model recovery over the unisensory model fits, and demonstrate that differences in priors and noise distributions can be successfully recovered through our model fitting and AIC/BIC pipeline. For the bisensory fits, the sheer number of candidate models and model optimization time had prohibited us from implementing exhaustive model recovery. Still, the fitting procedures mostly overlap between the unisensory and bisensory model fits, and hence the unisensory model recovery should be adequate in demonstrating the validity of model comparison.

Relevant information and a corresponding Fig 14 can be found in the Methods section of the revised manuscript, subsection "Model recovery analysis".

Introduction and discussion of the multisensory (causal inference) literature seem to rely on a subset of earlier publications. For example, the authors state that previous studies either assumed constant sensory noise across unisensory and multisensory conditions or did not use unisensory conditions at all. On the contrary, many modeling and non-modeling studies assume differences in noise between unisensory and multisensory conditions. Similarly, the authors discuss the uniqueness of their implementation of auditory remapping, but several cited and uncited studies have implemented remapping, i.e., miscalibrated sensory signals in their models.

We thank the Reviewer for pointing this out. We did not intend to suggest that our implementation of noise differences or recalibration was entirely novel—these ideas have precedent in the literature, which we have already cited [35, 22]; [15, 32, 33, 20, 28, 37, 12, 13, 10, 4]. If the Reviewer has specific additional references in mind beyond the ones in the current revision, we would be glad to include them.

Previous studies that modeled both multisensory tasks, bisensory localization and bisensory causal judgment, report that while localization responses are well described by non-vanilla causal inference models, direct causal inference judgments seem to rely on heuristic decision rules (Badde et al., 2019; Hong et al., 2021, 2022, 2025). This might explain why the study did not clearly select variants that implement model averaging, as most studies that favor model averaging only fit localization data.

We thank the Reviewer for raising this point. The Reviewer suggests an interesting possibility: that the inclusion of direct causal judgments (the BC task) in our fitting may have diluted evidence for model averaging, since such judgments may rely on heuristic strategies. This is consistent with our finding that MA and PM were difficult to distinguish, and warrants further investigation. We have incorporated this point into the Discussion:

> Fifth, although we varied the sensory noise and prior components in a semiparametric manner, the causal inference strategies used in the bisensory conditions could not be encoded as flexibly. Instead, we adopted several well-established strategies from the literature, including model selection, model averaging, and probability matching. Recent work has suggested that human causal inference may also rely on heuristic or rule-based mechanisms [5, 20, 21, 25, 1, 42], which we did not systematically examine here. Future research could aim to unify these diverse causal inference strategies within a semiparametric framework, which would enable a more systematic comparison of their descriptive power.

It is quite surprising to see that the locations of auditory stimuli influence the localization of high-reliability visual stimuli. Are participants' common cause priors very high? Had participants been told that visual and auditory stimuli would be presented in the same location half of the time?

For the best fitted bisensory model Exp-GaussianLaplace-PM, we have added a new Appendix section "Individual fitted parameters" with a corresponding Table S11. All participants have $p_{\text{same}} > 0.7$, which is even above the true probability of 0.5. This indeed suggests strong prior beliefs in the common-cause hypothesis.

Regarding participant instructions, they were not given information on the relative frequency of same- versus different-source trials. We now elaborate in the "Experiment procedure" subsection under Methods:

> (UV, UA): The instructions given to participants were: "Put your head in the chin rest to ensure your head is always in the same spot. Then look straight ahead and there will be a brief beep or a flash. Then use the mouse on the table to move the vertical line to the spot where you think it happened, and then click."
>
> (BV, BA): Participants were instructed: "There will be a flash and a beep at the same time. Use the mouse to indicate the location of the flash OR the beep, whichever the screen says at the beginning. Sometimes they will be in the same location and sometimes they won't." They were not given any information on the 50% same-location probability.
>
> (BC): Participants were instructed: "There will be a flash and a beep at the same time. Use the mouse to indicate the location of the flash OR the beep, whichever the screen says at the beginning. Sometimes they will be in the same location and sometimes they won't." They were not given any information on the 50% same-location probability.

Including a table with each participant's best-fitting parameters in the relevant model(s) (in the appendix) would be helpful.

We thank the Reviewer for suggesting this addition. For the best fitted bisensory model Exp-GaussianLaplace-PM, we have now added a new Appendix section "Individual fitted parameters" with a corresponding Table S11. For other models, each participant's fitted parameters are available in the Github repository.

Group-level averages can be misleading, despite the appeal of the curves. Some adjustments might help reassure the weary reader. Figures presenting summary statistics (e.g., bias or location-dependent SD) should show mean values and single participant statistics instead of standard errors of the mean.

We agree with the Reviewer's stance. Hence, the single-participant statistics were already visualized in the Appendix section "Model response distributions, individual participants".

If the noise distribution depends on location, is it reasonable to assume that auditory likelihoods—which cannot be inferred from population codes like in vision—are accurate?

This is an interesting question. Our models assume that auditory likelihoods reflect stimulus-dependent noise, which is a functional assumption that does not commit to a specific neural implementation. That said, there is evidence that the auditory system also features population coding [19, 11], which could in principle

support similar likelihood computations as in vision. A full treatment of neural implementation is beyond our current scope, but we agree this is an important direction for future work.

I am struggling to reconcile the recovered priors with the existing literature. Previous studies, especially those examining the perception of durations, demonstrate that participants quickly learn simple contextual priors, such as the uniform distributions used in this experiment. Why would participants in this study rely on a prior that does not match either the distribution of the stimuli or the typical visual and auditory locations encountered in real life? Why wouldn't they learn the contextual prior over five sessions?

We appreciate the Reviewer's questions. A key difference from prior-learning studies (such as those on duration perception [23, 3]) is that our participants received no feedback, and thus had no strong evidence for updating their beliefs toward the experimental distribution. Without error signals, it is not necessarily surprising that mismatched spatial priors persisted across sessions. We have added a note on this in the Discussion:

> In parallel to studies in higher-level cognition suggesting the flexibility of human priors for sophisticated events [16], it could be possible that similar top-down mechanisms are at play during human causal inference following initial perception [14]. Future work could clarify the environmental contexts that elicit specific prior assumptions, and investigate the optimality of such adjustments given potentially non-Gaussian statistics in the real world (especially in the absence of feedback as in the current experiment).

Minor comments

Reviewer comments about the style of a manuscript are subjective by nature, so whether or not the following comments are addressed is, in my view, the authors' creative freedom. To me, it seems unlikely that readers are able or willing to learn all of the acronyms the authors introduce and reliably retrieve them 20 pages later. I already failed at recalling them after a single paragraph. In my view, there is no good reason to force readers to learn new labels.

We apologize for the confusion created. We try to clarify the task-naming conventions at the beginning of the Methods section:

> Trials were organized in four types of blocks: *unisensory auditory localization* (UA), *unisensory visual localization* (UA), *bisensory localization* (B), and *bisensory causal judgment* (BC, where $C = 1$ corresponds to "same" and $C = 2$ correspond to "different"). The bisensory localization blocks contained interleaved trials of two response types (BV, BA), so there were five task types in total.

We now also include a separate Table 1 to clarify the model acronyms:

Table 1: Summary of models fitted. Numbers in parentheses convey the number of free parameters incurred by each modeling component (for sensory noise, the number of free parameters is multiplied by 2 due to separate $\boldsymbol{\theta}_{\mathrm{V}}, \boldsymbol{\theta}_{\mathrm{A}}$). The auditory range recalibration parameter is only systematically varied in the unisensory fits (*), and always free in bisensory fits. The semiparametric model is fitted only on unisensory data (**), while its fitted sensory noise and prior functions are then used for LiftedSemiparam models fitted to bisensory data (***). The acronyms MS, MA, and PM stand for model selection, model averaging, and probability matching causal inference strategies respectively.

**Model**	**Sensory noise**	**Prior**	**Aud. recalib. (unisensory*)**	**Causal inf. strategy (bisensory)**
Exp-GaussianLaplace	Exponential (3×2)	Gaussian-Laplace mixture (3)	1, 4/3, free (1)	MS, MA, PM
Exp-SingleGaussian	Exponential (3×2)	Gaussian (1)	free (1)	MS, MA, PM
Const-GaussianLaplace	Constant (1×2)	Gaussian-Laplace mixture (3)	free (1)	MS, MA, PM
Exp-TwoGaussians	Exponential (3×2)	Two-Gaussian mixture (3)	free (1)	MS, MA, PM
Const-SingleGaussian	Constant (1×2)	Gaussian (1)	1, 4/3, free (1)	MS, MA, PM
Semiparametric** LiftedSemiparam***	Flexible (12×2)	Flexible (11)	free (1)	MS, MA, PM

The manuscript is quite lengthy and sometimes reads more like a report. The authors might consider making the narrative more concise for the reader and relocating some material to the appendix.

We appreciate the Reviewer's suggestion, and understand the concern regarding the manuscript's length. We have aimed for clarity in describing the various model variants, as the paper's main contribution lies in systematic model comparison. Because these model distinctions are directly referenced throughout the Results section, we were compelled to introduce all model components in the main text for comprehension. We would welcome specific suggestions for parts to move to the Appendix if the Reviewer has particular sections in mind.

The manuscript makes several strong claims, which set a certain tone. I personally believe that dismissing others' work and ideas is neither necessary to demonstrate the quality of one's own work nor helpful in making science more welcoming.

We apologize if any language came across as dismissive—this was not our intention. Scientific progress involves identifying gaps in the literature, but we recognize this can be framed more or less generously. We have reviewed the manuscript and softened several passages where we felt our framing was too strong, including claims about what prior work did or did not do. We would welcome any additional specific suggestions if particular passages still strike the Reviewer as problematic.

References

[1] Acerbi, L., Dokka, K., Angelaki, D. E., and Ma, W. J. (2018). Bayesian comparison of explicit and implicit causal inference strategies in multisensory heading perception. *PLOS Computational Biology*, 14(7):1–38.

[2] Acerbi, L., Vijayakumar, S., and Wolpert, D. M. (2017). Target uncertainty mediates sensorimotor error correction. *PloS one*, 12(1):e0170466.

[3] Acerbi, L., Wolpert, D. M., and Vijayakumar, S. (2012). Internal representations of temporal statistics and feedback calibrate motor-sensory interval timing. *PLOS Computational Biology*, 8(11):1–19.

[4] Atkins, J. E., Jacobs, R. A., and Knill, D. C. (2003). Experience-dependent visual cue recalibration based on discrepancies between visual and haptic percepts. *Vision Research*, 43(25):2603–2613.

[5] Badde, S., Roeder, B., and Heed, T. (2019). Feeling a touch to the hand on the foot. *Current Biology*, 29(9):1491–1497.

[6] Beierholm, U. R., Quartz, S. R., and Shams, L. (2009). Bayesian priors are encoded independently from likelihoods in human multisensory perception. *Journal of Vision*, 9(5):23–23.

[7] Chakrabarti, A. and Ghosh, J. K. (2011). Aic, bic and recent advances in model selection. *Philosophy of statistics*, pages 583–605.

[8] Crane, B. T. (2012). Direction specific biases in human visual and vestibular heading perception. *PLOS ONE*, 7(12):1–15.

[9] Cuturi, L. F. and MacNeilage, P. R. (2013). Systematic biases in human heading estimation. *PLOS ONE*, 8(2):1–11.

[10] De Gelder, B. and Bertelson, P. (2003). Multisensory integration, perception and ecological validity. *Trends in Cognitive Sciences*, 7(10):460–467.

[11] Downer, J. D., Verhein, J. R., Rapone, B. C., O'Connor, K. N., and Sutter, M. L. (2021). An emergent population code in primary auditory cortex supports selective attention to spectral and temporal sound features. *Journal of Neuroscience*, 41(36):7561–7577.

[12] Ernst, M. O., Banks, M. S., and Bülthoff, H. H. (2000). Touch can change visual slant perception. *Nature Neuroscience*, 3(1):69–73.

[13] Ernst, M. O. and Bülthoff, H. H. (2004). Merging the senses into a robust percept. *Trends in Cognitive Sciences*, 8(4):162–169.

[14] Firestone, C. and Scholl, B. J. (2016). Cognition does not affect perception: Evaluating the evidence for "top-down" effects. *Behavioral and brain sciences*, 39:e229.

[15] Frissen, I., Vroomen, J., and de Gelder, B. (2012). The aftereffects of ventriloquism: the time course of the visual recalibration of auditory localization. *Seeing Perceiving*, 25(1):1–14.

[16] Griffiths, T. L. and Tenenbaum, J. B. (2006). Optimal predictions in everyday cognition. *Psychological science*, 17(9):767–773.

[17] Gu, Y., Fetsch, C. R., Adeyemo, B., Deangelis, G. C., and Angelaki, D. E. (2010). Decoding of MSTd population activity accounts for variations in the precision of heading perception. *Neuron*, 66(4):596–609.

[18] Hahn, M. and Wei, X.-X. (2024). A unifying theory explains seemingly contradictory biases in perceptual estimation. *Nature Neuroscience*, 27(4):793–804.

[19] Harper, N. S. and McAlpine, D. (2004). Optimal neural population coding of an auditory spatial cue. *Nature*, 430(7000):682–686.

[20] Hong, F., Badde, S., and Landy, M. S. (2021). Causal inference regulates audiovisual spatial recalibration via its influence on audiovisual perception. *PLOS Computational Biology*, 17(11):1–37.

[21] Hong, F., Badde, S., and Landy, M. S. (2022). Repeated exposure to either consistently spatiotemporally congruent or consistently incongruent audiovisual stimuli modulates the audiovisual common-cause prior. *Scientific reports*, 12(1):15532.

[22] Huang, N. and Elhilali, M. (2020). Push-pull competition between bottom-up and top-down auditory attention to natural soundscapes. *eLife*, 9:e52984.

[23] Jazayeri, M. and Shadlen, M. N. (2010). Temporal context calibrates interval timing. *Nature Neuroscience*, 13(8):1020–1026.

[24] Körding, K. P., Beierholm, U., Ma, W. J., Quartz, S., Tenenbaum, J. B., and Shams, L. (2007). Causal inference in multisensory perception. *PLOS ONE*, 2(9):1–10.

[25] Li, L., Hong, F., Badde, S., and Landy, M. S. (2025). Precision-based causal inference modulates audiovisual temporal recalibration. *eLife*, 13:RP97765.

[26] Ma, W. J., Kording, K. P., and Goldreich, D. (2023). *Bayesian models of perception and action: An introduction*. MIT press.

[27] Natarajan, R., Murray, I., Shams, L., and Zemel, R. (2009). Characterizing response behavior in multisensory perception with conflicting cues. In Koller, D., Schuurmans, D., Bengio, Y., and Bottou, L., editors, *Advances in Neural Information Processing Systems 21*, pages 1153–1160.

[28] Odegaard, B., Wozny, D. R., and Shams, L. (2015). Biases in visual, auditory, and audiovisual perception of space. *PLOS Computational Biology*, 11(12):1–23.

[29] Parise, C. V. (2024). A stimulus-computable model for audiovisual perception and spatial orienting in mammals. *bioRxiv*, pages 2023–12.

[30] Polanía, R., Woodford, M., and Ruff, C. C. (2019). Efficient coding of subjective value. *Nature neuroscience*, 22(1):134–142.

[31] Prat-Carrabin, A. and Woodford, M. (2022). Efficient coding of numbers explains decision bias and noise. *Nature Human Behaviour*, 6(8):1142–1152.

[32] Recanzone, G. H. (1998). Rapidly induced auditory plasticity: The ventriloquism aftereffect. *Proceedings of the National Academy of Sciences*, 95(3):869–875.

[33] Recanzone, G. H. (2009). Interactions of auditory and visual stimuli in space and time. *Hear Res*, 258(1-2):89–99.

[34] Sato, Y., Toyoizumi, T., and Aihara, K. (2007). Bayesian inference explains perception of unity and ventriloquism aftereffect: Identification of common sources of audiovisual stimuli. *Neural Computation*, 19(12):3335–3355.

[35] Talsma, D., Senkowski, D., Soto-Faraco, S., and Woldorff, M. G. (2010). The multifaceted interplay between attention and multisensory integration. *Trends Cogn Sci*, 14(9):400–410.

[36] Vandekerckhove, J., Matzke, D., and Wagenmakers, E.-J. (2015). 14 model comparison and the principle of parsimony. *The Oxford handbook of computational and mathematical psychology*, page 300.

[37] Watson, D. M., Akeroyd, M. A., Roach, N. W., and Webb, B. S. (2021). Multiple spatial reference frames underpin perceptual recalibration to audio-visual discrepancies. *PLOS ONE*, 16(5):1–21.

[38] Wei, X.-X. and Stocker, A. A. (2015). A bayesian observer model constrained by efficient coding can explain'anti-bayesian'percepts. *Nature neuroscience*, 18(10):1509–1517.

[39] Wilson, R. C. and Collins, A. G. (2019). Ten simple rules for the computational modeling of behavioral data. *elife*, 8:e49547.

[40] Wozny, D. R., Beierholm, U. R., and Shams, L. (2008). Human trimodal perception follows optimal statistical inference. *Journal of Vision*, 8(3):24–24.

[41] Wozny, D. R., Beierholm, U. R., and Shams, L. (2010). Probability matching as a computational strategy used in perception. *PLOS Computational Biology*, 6(8):1–7.

[42] Zhou, Y., Acerbi, L., and Ma, W. J. (2020). The role of sensory uncertainty in simple contour integration. *PLoS computational biology*, 16(11):e1006308.
